# Chemokine Regulation During Epidemic Coronavirus Infection

**DOI:** 10.3389/fphar.2020.600369

**Published:** 2021-02-04

**Authors:** Shamik Majumdar, Philip M. Murphy

**Affiliations:** Molecular Signaling Section, Laboratory of Molecular Immunology, National Institute of Allergy and Infectious Diseases, NIH, Bethesda, MD, United States

**Keywords:** SARS-CoV, SARS-CoV-2, MERS-CoV, SARS, MERS, COVID-19

## Abstract

SARS-CoV-2 (Severe Acute Respiratory Syndrome coronavirus-2) is the third coronavirus to emerge as a cause of severe and frequently fatal pneumonia epidemics in humans, joining SARS-CoV and MERS-CoV (Middle East Respiratory Syndrome-coronavirus). As with many infectious diseases, the immune response to coronavirus infection may act as a double-edged sword: necessary for promoting antiviral host defense, but, if not appropriately regulated, also able to incite life-threatening immunopathology. Key immunoregulatory mediators include the chemokines, a large family of leukocyte chemoattractants that coordinate leukocyte infiltration, positioning and activation in infected tissue by acting at specific G protein-coupled receptors. Here, we compare the involvement of chemokines and chemokine receptors during infection with the three epidemic coronaviruses and discuss their potential value as biomarkers and targets for therapeutic development.

## Introduction

The emergence and re-emergence of infectious agents in populations lacking pre-existing immunity is a bane of the human condition that has only worsened since the industrial revolution, primarily due to population growth, urban concentration, wild habitat encroachment and advances in transportation. Since the late 1960s when the antibiotic and vaccine revolutions prompted the US Surgeon General William H. Stewart to prematurely declare the end of infectious diseases (Spellberg, 2008), at least 87 microorganisms have emerged as serious human pathogens, causing either small outbreaks, epidemics or pandemics with highly diverse mortality rates (Woolhouse and Gaunt, 2007). Three of the pathogens that emerged during this period were coronaviruses, a previously unimportant class of organisms in the history of infectious diseases of man. SARS-CoV was the first coronavirus to cause an epidemic of fatal respiratory infection in humans, SARS, which lasted from 2002 to 2004. This was followed in 2012 by MERS-CoV, the cause of the MERS pandemic, and in 2019 by SARS-CoV-2, the cause of the ongoing COVID-19 pandemic. Before 2002, four other coronaviruses (229E, HKU1, NL43 and OC43) had been described as human pathogens; however, all four cause only mild respiratory illness, predominantly the common cold.

All coronaviruses are spherical in shape and ∼0.1 microns in diameter. An outer lipid envelope encloses an ∼30 kb nonsegmented, single-stranded, positive-sense RNA genome that encodes 4 structural and 16 nonstructural proteins (nsps). The structural proteins include Spike (S), Membrane (M), Envelope (E) and Nucleocapsid (N). Spike projects from the lipid envelope and studs the entire surface of the virus, giving it the appearance of the corona of the sun, thus the name ‘coronavirus’. Coronaviruses are classified into 4 genera: alphacoronavirus, betacoronavirus, gammacoronavirus, and deltacoronavirus.

SARS-CoV, MERS-CoV and SARS-CoV-2 are all betacoronaviruses (Coronaviridae Study Group of the International Committee on Taxonomy of Viruses, 2020). All three appear to have jumped from bat reservoirs to humans, either directly or indirectly through an intermediate mammalian host. Bats make up ∼25% of all mammalian species and are a major reservoir of zoonotic pathogens. Many other coronaviruses have been described that naturally infect other mammals, including cats, dogs, civets, pigs, mice, ferrets, mink, camels, horses, pangolins, non-human primates and whales. Factors favoring emergence include the low fidelity of the viral RNA-dependent-RNA polymerase, a high frequency of RNA recombination, increasing human-animal reservoir interactions, broad host species tropism and the high prevalence of coronaviruses (Cui et al., 2019). Thus, opportunities for spillover expand as human populations encroach on bat habitat, which may be advantaged by an opportunistic coronavirus as it rapidly adapts to the new intermediate or accidental human host through serial passage, thanks to a faulty polymerase. Although all three epidemic coronaviruses are highly pathogenic, there is a striking rank order for mortality, MERS>SARS>>SARS-CoV-2, which is the inverse of the rank orders for both transmissibility and global disease burden, SARS-CoV-2>>>SARS>MERS (Petersen et al., 2020a).

The epidemic coronaviruses typically infect by an intranasal, oral or conjunctival route, and produce diverse outcomes ranging from asymptomatic infection to severe pneumonia, acute respiratory distress syndrome (ARDS), multiorgan failure and death. Virus-triggered inflammation is necessary for viral clearance and for initiation of an adaptive immune response. However, an unchecked and dysregulated immune response, characterized by high levels of inflammatory cytokines known as a ‘cytokine storm’, may produce more severe outcomes (Matthay et al., 2019). Cytokine storm is a general and scientifically imprecise term that may vary in molecular pathogenesis depending on the trigger. For example, the molecular mechanisms in bacterial sepsis may include different elements from those driving severe COVID-19, even though the anatomic pathologic features and clinical consequences may have extensively overlapping features. ARDS results from inexorable flooding of the alveolar lumen with proteinaceous edema fluid containing abundant fibrin and inflammatory cells, which reduce alveolar gas exchange.

The FDA has authorized the use of two mRNA-based vaccines developed by Moderna and Pfizer for prevention of COVID-19. The viral polymerase blocker remdesivir for COVID-19, which was reported to reduce the time to discharge for hospitalized patients by four days but without a clear effect on mortality (Beigel et al., 2020) has also been approved by the FDA for treatment of coronavirus infection. Subsequently, the monoclonal antibodies, Bamlanivimab, Casirivimab and Imdevimab which target the receptor binding domain of the spike protein of SARS-CoV-2, as well as Baricitinib, a Janus kinase inhibitor in combination with Remdesivir, have also been approved by the FDA for treatment of COVID-19 at various stages of the disease. Many other agents are currently under evaluation in observational and controlled clinical trials worldwide, including both direct antiviral agents and immune modulators. Immunological targets for development have not been validated yet; however, the list of candidates is long, motivated by data from preclinical and clinical studies of immune system function and specific antiviral response pathways. Among the many potential targets, chemokines have attracted substantial interest, owing in part to the high levels that have been measured in patients presenting with severe disease.

Chemokines are a large family (n∼45 in human) of small secreted cytokines that coordinate leukocyte trafficking and activation, thereby regulating diverse physiological processes, including development, inflammation, immune responses and wound healing. Based on the number and spacing of conserved cysteine residues near the *N*-termini, chemokines are classified into four subfamilies: CXC, CC, CX3C and C (Griffith et al., 2014). Chemokines act at G protein-coupled receptors (GPCRs), a rich target for drug development in general, and one which has been interrogated broadly by the pharmaceutical industry in the case of chemokine receptors, with many blocking agents now in clinical trials for diverse disease indications. Like all GPCRs, the 18 known human chemokine GPCRs have seven transmembrane domains and signal via heterotrimeric G proteins, Table 1 (Griffith et al., 2014). Four atypical chemokine receptors that do not signal through G proteins have also been described. Chemokines can be divided loosely into two main functional groups depending on whether they support mainly homeostatic or inflammatory immune processes. During acute viral pneumonia, many inflammatory chemokines are upregulated by infected and activated cells to facilitate effector leukocyte recruitment to sites of infection, promoting inflammation, immune cell activation, clearance of the viral pathogen and, in severe cases, immunopathology (Glass et al., 2003). Local and systemic induction of chemokines during many types of acute viral pneumonia has been directly correlated with disease severity in humans, and in some examples specific chemokines and chemokine receptors have been demonstrated to play important roles in disease pathogenesis (Glass et al., 2003; Castelli et al., 2020; Coperchini et al., 2020).

**Table 1:** General functions of chemokines and chemokine receptors that are differentially regulated in lung during acute coronavirus infections in humans and animal models.

Subfamily	Systematic name	Alternative name/s	Receptor/s	Functions
CC	CCL1	I-309	CCR8	Type 2 immune responses and regulatory T cell trafficking
	CCL2	MCP-1	CCR2	Inflammatory monocyte trafficking, Type 1 immune responses
	CCL3	MIP-1α	CCR1/CCR5	Macrophages and NK cell trafficking; Type 1 immune responses
	CCL4	MIP-1β	CCR5	T cell-DC interaction; Type 1 immune responses
	CCL5	RANTES	CCR1/CCR3/CCR5	Type 1 and 2 immune responses
	CCL7	MCP-3	CCR2/CCR3	Monocyte trafficking
	CCL8	MCP-2	CCR1/CCR2/CCR3/CCR5 (human)/CCR8 (mouse)	Type 2 immune response; T cell homing to skin
	CCL11	Eotaxin-1	CCR3	Eosinophil and basophil trafficking
	CCL17	TARC	CCR4	Type 2 immune responses; Treg function; homing of T cells to lung and skin
	CCL20	MIP-3α	CCR6	Th17 responses, B and DC homing in gut-associated lymphoid tissue
	CCL24	Eotaxin-2	CCR3	Eosinophil and basophil trafficking
	CCL27	CTAK	CCR10	T cell homing to skin and mucosal sites
CXC	CXCL1	GROα, KC (mouse)	CXCR2	Neutrophil trafficking
	CXCL2	GROβ, MIP-2α, MIP-2 (mouse)	CXCR2	Neutrophil trafficking
	CXCL8	IL-8 (not in mice)	CXCR1/CXCR2	Neutrophil trafficking
	CXCL9	MIG	CXCR3	Type 1 immune responses
	CXCL10	IP-10	CXCR3	Type 1 immune responses
	CXCL11	I-TAC	CXCR3	Type 1 immune responses
C	XCL1	Lymphotactin α	XCR1	Cross presentation by CD8^+^ DCs
	XCL2	Lymphotactin β	XCR1	Cross presentation by CD8+ DCs
CX3C	CX_3_CL1	Fractalkine	CX3CR1	NK, T cells and monocyte trafficking

CTAK, Cutaneous T cell-attracting chemokine; GRO, Growth-regulated oncogene; I-TAC, Interferon-inducible T-cell alpha chemoattractant; IP-10, Interferon gamma-induced protein 10; KC, Keratinocyte-derived chemokine; MCP-1, Monocyte chemoattractant protein-1; MIG, Monokine induced by gamma interferon; MIP, Macrophage inflammatory protein; RANTES, Regulated upon activation, normal T cell expressed and secreted; TARC, thymus and activation regulated chemokine, Treg, Regulatory T cells. Table adapted from (Griffith et al., 2014).

A comprehensive understanding of the host response during any infection may inform efforts to develop targeted anti-inflammatory treatments that might complement direct antimicrobial agents in severe cases. Given the staggering global disease and socioeconomic burden of epidemic coronavirus infection, it is crucial to fully understand host-pathogen interactions in the lung, the primary site of infection. Already, with the absence of any deep understanding, the general and powerful immunosuppressant dexamethasone has shown some efficacy in the treatment of patients with severe COVID-19. Since the COVID-19 pandemic is still in its first year and its immunopathogenesis remains poorly defined, we have compared what is currently known about the chemokine system in all three epidemic coronavirus infections, with the anticipation that there may be shared features able to guide and accelerate studies of the chemokine system in the pandemic that may inform novel ways to suppress immunopathology and the cytokine storm.

### SARS-CoV

Like MERS-CoV and SARS-CoV-2, SARS-CoV originated in bats (Hu et al., 2017). It was introduced into humans in China in 2002 by spillover from an intermediate host, most likely palm civets, then spread to 29 countries, including the United States and Canada (Peiris et al., 2003b; Ksiazek et al., 2003; Cui et al., 2019). More than 8,000 cases were reported before the epidemic was controlled in 2004 by the classic epidemiologic containment measures of case identification, contact screening and quarantine (https://www.who.int/csr/sars/country/2003_07_11/en/, last accessed on August 17, 2020). The impact of SARS on emerging infectious disease awareness and pandemic preparedness was profound, driven by its high overall case-fatality rate of almost 10%.

The SARS incubation period is 2–7 days. Asymptomatic and presymptomatic transmission does not appear to be significant. Instead, patients typically display characteristic but non-specific clinical manifestations, including fever, dry cough, headache, dyspnea and myalgia, resulting in person to person transmission via respiratory droplets and aerosols. Epidemiologists described both symptomatic superspreader events during the SARS epidemic as well as evidence for remote transmission, as documented most clearly through air vents in the Metropole hotel in Hong Kong (Tsang et al., 2003). Severe disease is characterized by recurrent fever, oxygen desaturation and pneumonia, which frequently progresses to ARDS requiring mechanical ventilatory support (Booth et al., 2003; Lee et al., 2003; Peiris et al., 2003a; Peiris et al., 2003b). Clinical factors predictive of poor outcome and death in SARS include advanced age, male sex, neutrophilia, lymphopenia, superinfection and comorbidities such as diabetes (Peiris et al., 2003a; Peiris et al., 2003b; Booth et al., 2003; Choi et al., 2003; Lee et al., 2003; Chen et al., 2005; Jiang et al., 2005; Tang et al., 2005; Chien et al., 2006).

#### SARS Immunopathogenesis

Peak SARS-CoV viral burden precedes SARS disease progression, which is associated with a declining absolute lymphocyte count in the blood, rising serum lactate dehydrogenase levels, and progression of lung infiltrates on radiographs (Peiris et al., 2003a; Wang et al., 2004). SARS-CoV was reported as undetectable in the lungs of patients who died more than two weeks after showing signs of illness (Nicholls et al., 2006), suggesting that fatal disease progression may become independent of the virus and instead may be driven by dysregulated immunopathology.

In severe and fatal cases of SARS, the infected lung displays diffuse alveolar damage (DAD), consolidation, inflammatory cell infiltration, hyaline membrane formation (fibrin deposition with remnants of necrotic epithelial cells) and multinucleated giant cells derived from epithelial cells or macrophages (Choi et al., 2003; Franks et al., 2003; Ksiazek et al., 2003; Lee et al., 2003; Nicholls et al., 2003; Tse et al., 2004; Gu et al., 2005; He et al., 2006). Lung autopsies of SARS patients suggest that productive infection initially occurs in alveolar epithelial cells followed by macrophages (Gu et al., 2005; Nicholls et al., 2006; Mossel et al., 2008). At least two host cell membrane factors have been identified that facilitate SARS-CoV entry. Angiotensin-converting enzyme-2 (ACE2) is a metallopeptidase used as the cellular SARS-CoV receptor (Li et al., 2003; Hoffmann et al., 2020; Wan et al., 2020). It is also used by SARS-CoV-2, but not by MERS-CoV. Accordingly, in lung ACE2 is expressed on alveolar and bronchial epithelial cells, bronchial serous glands and alveolar macrophages (He et al., 2006). Poor usage of mouse ACE2 by SARS-CoV was a limitation to mouse models of SARS that was overcome by developing transgenic (Tg) mice expressing human ACE2 (hACE2) (Table 2). Transmembrane serine protease 2 (TMPRSS2) is a second host cell surface molecule that augments SARS-CoV and MERS-CoV infectivity by cleaving both ACE2, which promotes viral uptake, and the viral S protein, which activates S protein for membrane fusion. Accordingly, mice deficient in TMPRSS2 display reduced severity of disease following experimental SARS-CoV and MERS-CoV infection, including decreased lung pathology and dampened chemokine induction (Iwata-Yoshikawa et al., 2019a) (Tables 2 and 3).

**Table 2:** Chemokine modulation in lungs of animal models of SARS-CoV infection.

Animal model	Virus modification	Manifestations	Chemokine modulation in lungs	Reference
Mice	Wild-type	Mild weight loss, mild lung inflammation with no cell infiltration	Upregulation of Ccl1, Ccl2, Ccl3, Ccl5, Cxcl1, Cxcl9, Xcl1, Cxcl10, Ccr1 and Cxcr3; Ccr3 was downregulated	Glass et al., (2004)
Aged mice	Wild-type	Weight loss, alveolar damage, infiltration of NK cells, macrophages, plasmacytoid DCs and T cells	Biphasic protein induction of CCL2, CCL3, CCL5, CXCL9 and CXCL10;>Induction of Ccr2, Ccr5 and Cxcr3	Chen et al., (2010b)
Mice	Adapted	Excessive weight loss, pulmonary inflammation and consolidation, and mortality	CXCL10 upregulation	Kumaki et al., (2010)
Mice	Adapted	Weight loss, moderate perivascular edema and infiltrates, highly lethal	CCL2, CCL3 and CCL5 upregulation	Day et al., (2009)
Young and aged mice	Adapted	Enhanced weight loss, pulmonary edema, fibrin deposition and hyaline membrane formation, neutrophil and inflammatory macrophage infiltration, fatality in aged mice as compared to young mice	Upregulation of CCL2, CCL3, KC, CXCL9 and CXCL10 with varying kinetics in young and old mice	Nagata et al., (2008)
Middle aged male and female mice	Adapted	Enhanced alveolar edema and terminal bronchiolar epithelial sloughing and greater mortality in male mice than age-matched female mice	Prolonged and heightened Ccl2 and Cxcl1 induction in male mice compared to female mice	Channappanavar et al., (2017)
Mice	Adapted lacking the viral E protein	Reduced lung viral titer, neutrophil infiltrates in lungs, and pulmonary damage; absence of weight loss and mortality as compared to WT virus	Reduced induction of Ccl2, Ccl5, Cxcl1, Cxcl2 and Cxcl10 by the mutant virus compared to the WT virus	DeDiego et al., (2014)
Mice	Adapted lacking/defective viral E protein-PDZ binding motif	Reduced weight loss and mortality compared to the WT strain; decreased viral burden in lung; reduced interstitial and peribronchial cell infiltration and alveolar and bronchiolar edema	Reduced induction of Ccl2 and Cxcl10 by the mutant viruses compared to the WT virus	Jimenez-Guardeño et al., (2014)
MyD88 KO mice	Adapted	Weight loss, denuding bronchiolitis, delayed recruitment of inflammatory monocytes/macrophages in lung and mortality	Reduced induction of Ccl2, Ccl3 and Ccl5 by the mutant virus compared to the WT mice.	Sheahan et al., (2008)
Mice - hACE2 under the cytokeratin 18 promoter	Wild-type	Weight loss, perivascular and peribronchiolar inflammation, inflammatory cell infiltrates and mortality	Induction of Ccl2, Ccl7, Cxcl9 and Cxcl10	McCray et al., (2007)
Mice - hACE2 under CAG promoter^a^	Wild-type	Weight loss, interstitial pneumonitis and inflammatory infiltrates and mortality	Upregulation of KC, CCL2 and CCL5 amounts.	Tseng et al., (2007); Yoshikawa et al., (2009a)
TMPRSS2 KO mice	Adapted	Reduced weight loss, focal inflammatory infiltration in alveoli, non-lethal infection	Reduced amounts of CCL2, CCL3, KC, CXCL9 and CXCL10 in lungs of infected KO mice compared to WT mice	Iwata-Yoshikawa et al., (2019a)
STAT1 KO mice	Wild-type	Weight loss, pulmonary edema, neutrophil, lymphocyte, macrophage and eosinophil infiltration, mortality	Increased CCL2 levels in lungs as compared to WT mice	Frieman et al., (2010)
Adolescent cynomolgus macaques	Wild-type	Alveolar edema, infiltration of neutrophils, non-lethal	Induction of *CXCL8* and *CXCL10*	de Lang et al., (2007)
Young and aged cynomolgus macaques	Wild-type	Enhanced multifocal pulmonary lesions and leukocyte infiltration in aged animals	Higher induction of *CXCL8* and *CCL20* in aged macaques	Smits et al., (2010); Smits et al., (2011)
African green monkeys	Wild-type	Multifocal pulmonary consolidation, DAD and moderate leukocyte infiltration	Upregulation of *CCL3* and *CCL20* but not *CXCL8*.	Smits et al., (2011)

^a^ A composite promoter composed of the cytomegalovirus immediate-early enhancer, the chicken β-actin promoter and the rabbit globin splicing and polyadenylation site.

**Table 3:** Chemokine regulation in the lungs of animal models of MERS-CoV-induced viral pneumonia.

Animal model	Virus modification	Manifestations	Chemokine modulation in lungs	Reference
Mice - hDPP4 under the CAG promoter	Wild-type	Weight loss, multifocal consolidation, macrophage and lymphocyte infiltration and death	Upregulation of Ccl2, Ccl3, Ccl5, Cxcl1 and Cxcl10	Agrawal et al., (2015)
Mice - hDPP4 under cytokeratin 18 promoter	Wild-type	Weight loss, patchy consolidation and death	Upregulation of Ccl2, Cxcl9 and Cxcl10	Li et al., (2016)
Mice - hDPP4 knockin Tg mice	Adapted	Fatal pneumonia and infiltration of macrophages and neutrophils in lungs	Upregulation of Ccl2, Cxcl9 and Cxcl10 expression and increased amounts of CCL2, KC, MIP-2 and CXCL10	Li et al., (2017)
Mice - hDPP4 under an endogenous mouse DPP4 promoter	Wild-type	Mild, non-lethal infection	Elevated CCL2, CCL3, CXCL9 and CXCL10 levels	Iwata-Yoshikawa et al., (2019b)
Mice - hDPP4 under an endogenous DPP4 promoter and KO for TMPRSS2	Wild-type	Mild, slight weight loss, mild mononuclear alveolar infiltration, non-lethal infection	Delayed induction of CCL2, CCL3, and CXCL9 amounts in TMPRSS2-hDPP4 mice as compared to hDPP4 mice	Iwata-Yoshikawa et al., (2019a)
Mice - hDPP4 under an endogenous DPP4 promoter, Partial depletion of CD8 T cells	Wild-type	Dose-dependent, asymptomatic to lethal infection; infiltration of T cells, macrophages and neutrophils; Reduced CD8 T cells ameliorates bronchiolar inflammation, lymphocyte infiltration and pleuritis	Upregulation of Ccl2, Ccl7, Ccl12, Cxcl9, Cxcl10 and Cxcl11 (RNA-seq)	Coleman et al., (2017)
Common marmoset	Wild-type	Bronchointerstitial pneumonia, with lethality in some animals	Alterations in chemokine-related pathways detected by RNA-seq	Falzarano et al., (2014)
Rhesus macaques	Wild-type	Interstitial infiltration and multifocal consolidation	Elevated CCL2 levels reduced by interferon-α2b and ribavirin treatment	Falzarano et al., (2013)

Lung autopsies of SARS patients and *in vitro* SARS-CoV infections of macrophages and alveolar and bronchial cells have clearly demonstrated upregulation of numerous monocyte/macrophage, neutrophil and T cell-specific chemokines, including CCL2, CCL5, CXCL8, CXCL9 and CXCL10 (Yen et al., 2006; Yoshikawa et al., 2010). In agreement with this, the autopsies revealed infiltration of macrophages, neutrophils and T cells, but not B or NK cells (Hsueh et al., 2004; Gu et al., 2005; He et al., 2006; Yen et al., 2006). Moreover, peripheral blood levels of the same inflammatory chemokines have been associated with adverse outcomes in SARS patients (Huang et al., 2005; Tang et al., 2005; Chien et al., 2006; Cameron et al., 2007). Elevated levels of CXCL10 in plasma early in infection have been reported to be a particularly poor prognostic indicator (Tang et al., 2005; Chien et al., 2006).

At the histologic level, strong local CCL2 expression in infected ACE2^+^ alveolar and bronchial epithelial cells (He et al., 2006) as well as increased CXCL10 expression have been observed in lung samples obtained at autopsy (Jiang et al., 2005; Tang et al., 2005; Danesh et al., 2008). Consistent with this, CXCR3, the receptor for CXCL10 (as well as CXCL9 and CXCL11), was strongly upregulated in lung samples obtained at autopsy from SARS patients (Danesh et al., 2008). CXCL10 expression peaks in lung during early stages of disease progression, whereas the monocyte-macrophage and T cell-directed chemokine CCL3, the T cell chemokine CCL27, and the neutrophil-targeted chemokines CXCL2 and CXCL8 are upregulated in lung during late stages of disease (Kong et al., 2009). SARS-CoV can also infect primary human monocyte-derived macrophages (MDMs) to induce the expression of CCL2 and CXCL10 (Cheung et al., 2005), and can infect human dendritic cells (DCs) to induce the expression of CCL2, CCL3, CCL5 and CXCL10 (Law et al., 2005), as well as the receptors CCR1, CCR3 and CCR5 (Law et al., 2009). However, viral replication is non-productive in both cell types. Further, *in vitro* infection of human type II alveolar epithelial cells maintained at an air-liquid interface led to productive virus replication and significant upregulation of CXCL10 and CXCL11, as well as CXCL8 and the Th1 cell-directed chemokine CCL5 (Qian et al., 2013). In additional cell-based studies, SARS-CoV infection induced expression of CXCL8 and CCL2 in A549 lung epithelial cells and THP-1 monocytic cells (Yen et al., 2006). Compared with the less virulent coronavirus CoV-229E, SARS-CoV induced higher levels of inflammatory chemokines in both A549 and THP-1 cells. Consistent with this, supernatants from SARS-CoV-infected cells showed chemotactic activity towards neutrophils (CXCL8-dependent), monocytes (CCL2- and CCL5-dependent) and activated T cells (CXCL8-, CCL2- and CCL5-dependent) (Yen et al., 2006).

In mice infected with SARS-CoV, a similar pattern of inflammatory chemokine induction occurs as in infected human cells and tissue. Moreover, infection with lethal strains of SARS-CoV demonstrated worse lung pathology and higher levels of induction of inflammatory chemokines, such as Cxcl10 and Ccl2, than infection with non-lethal SARS-CoV strains that replicated to similar or even higher levels in the lung (Rockx et al., 2009). Microarray studies in infected ferrets have also documented upregulation of *CCL2* and *CXCL10* in lung. The effect is limited to primary infection and does not occur after reinfection. The authors of the study suggest that adaptive immunity restricts viral replication during reinfection, thus limiting the induction of the innate immune response. Therefore, the innate responses may be required only during the acute phase of infection (Cameron et al., 2012).

Overall, a model has emerged in which SARS-CoV primarily infects lung epithelial cells to undergo replication, followed by infection in macrophages, with induction of chemokine expression in both cell types. Next, chemokines mediate recruitment of additional macrophages, neutrophils and T cells. Upon activation, these leukocytes contribute to an exuberant immune response which may involve further production of chemokines, potentially contributing to immunopathological damage in the lung and development of ARDS.

#### Direct and Indirect Chemokine Regulation by SARS-CoV

Vaccination with SARS-CoV structural proteins can independently influence chemokine expression after viral challenge. In particular, in >6 month-old mice immunized intradermally with recombinant vaccinia viruses encoding M, N or E, subsequent challenge with SARS-CoV resulted in upregulation of Ccl2, Ccl3 and Cxcl10 in the lung. These chemokines were not significantly upregulated in the lungs of infected mice vaccinated with vector alone or with recombinant vaccinia virus encoding the SARS-CoV S protein. Vaccination with S protein, but not with M, N and E proteins expressed independently, protected mice from subsequent SARS-CoV challenge, as evidenced by reduced viral titers in the lung (Yasui et al., 2008). Purified SARS-CoV S protein stimulation of the human lung epithelial cell line A549 was capable of upregulating CCL2 (Chen et al., 2010a). Expression of SARS-CoV S by plasmid transfection could also upregulate CXCL8 (Chang et al., 2004) in human lung epithelial cell lines A549 and NCI-H520 and human lung fibroblast cell lines HFL-1 and MRC-5. SARS-CoV S-mediated chemokine upregulation in both these studies was dependent on MAP kinase, but independent of NF-κB activity.

E protein-deficient SARS-CoV is less pathogenic and demonstrates diminished NF-κB activation in infected mouse lung (Table 2). Moreover, direct pharmacologic inhibition of NF-κB by the inhibitors caffeic acid phenethyl ester/CAPE and parthenolide *in vivo* reduces expression of *Ccl2* and *Cxcl2* associated with less pulmonary damage and mortality in mice, without affecting viral burden in the lungs (DeDiego et al., 2014). In A549 cells, plasmid-encoded SARS-CoV nonstructural protein nsp1 induced CCL3, CCL5 and CXCL10 expression in an NF-κB-dependent manner. Similarly expressed nsp1 from the coronavirus mouse hepatitis virus and the less pathogenic human coronaviruses HCoV-229E and HCoV-OC43 were unable to significantly induce the expression of the aforementioned chemokines in these systems (Law et al., 2007).

SARS-CoV is well-known to repress type I interferon (IFN) expression, which in turn may influence chemokine expression. This can be seen clearly, for example, *in vitro*, where IFN-α pretreatment of SARS-CoV-infected human HEK 293 cells induces overexpression of CXCL10 and CXCL11 relative to uninfected IFN-α-stimulated cells (Kuri et al., 2009), whereas in the absence of exogenous IFN stimulation SARS-CoV infection is unable to induce IFN-β as well as CXCL10 and CXCL11 expression. *In vivo*, reports of disproportionately sparse infiltration of inflammatory cells in the lungs of both fatal SARS cases (Lee et al., 2003; Tse et al., 2004; Gu et al., 2005) and SARS-CoV-infected African green monkeys (Smits et al., 2011) may be associated with virus-induced repression of type I IFN and type I IFN-inducible chemokines. Consistent with this, serum CXCL8 was not elevated in SARS patients, while being highly elevated in community-acquired pneumonia (CAP) patients (Chien et al., 2006). The apparent discrepancy between this study, where CXCL8 amounts in sera of SARS patients remained uninduced, and reports mentioned in the previous sections where CXCL8 levels in lungs and lung cell lines were an indicator of severity of SARS, might reflect differences between systemic and local lung induction.

Timely type I IFN signaling is crucial for controlling SARS-CoV viral load, and for resolution of lung pathology in mice. Administration of IFN-β 6 h prior to the peak of infection, but not 12 and 24 h post infection, protected WT mice against lethal infection (Channappanavar et al., 2016). Likewise, in SARS-CoV-infected aged macaques, administration of pegylated IFN-α intramuscularly on days 1 and 3 post infection ameliorated lung pathology and dampened *CXCL8* induction without affecting viral burden in the lung (Smits et al., 2010). In fact, both loss and gain of type I IFN signaling can be protective in the model. Mice lacking the IFNαβ receptor (IFNAR) are protected against lethal infection by a mouse-adapted (MA) strain of SARS-CoV. Compared to WT mice, infected *Ifnar* ^*−/−*^ mice had similar viral burden in lung tissue, but only transient weight loss, less alveolar edema, increased peribronchial-perivascular immune cell infiltration, and either delayed or absent expression of IFNβ, Ccl2 and pro-inflammatory cytokines in the lung. On the other hand, in infected mice, type I IFN signaling promoted Ccl2 expression in lung associated with the local influx of highly activated inflammatory monocyte-macrophages, a source of Ccl2 and other pro-inflammatory molecules. Depletion of these cells by anti-Ccr2 antibody (inflammatory monocytes specifically express Ccr2) reduced the levels of Ccl2 in bronchoalveolar lavage fluid (BALF) and reduced lung pathology and mortality, all of which were independent of the viral load in the lung (Channappanavar et al., 2016). For the virus, IFN-I suppression may be useful to allow for efficient viral propagation prior to inducing a cytokine storm. Eventually, SARS-CoV proteins override the relative lack of type I IFN observed during the initial phases of infection to induce chemokine gene expression, possibly to infect immune cells including macrophages and T cells (Gu et al., 2005) and to further enhance the cytokine cascade.

Timely induction and waning of chemokine expression may be important for resolution of SARS. Serum levels of the anti-inflammatory cytokine IL-10 were inversely proportional to CCL2, CXCL9 and CXCL10 in SARS patients (Huang et al., 2005). At convalescent stages, IL-10 levels were observed to be significantly elevated (Zhang et al., 2004). However, a conflicting report claimed IL-10 levels remained uninduced at all stages of SARS but were significantly induced at progressive and worse stages of CAP (Chien et al., 2006).

Lymphopenia may occur in severe SARS disease and may predispose patients to secondary infections and increased mortality. SARS patients with secondary *Pseudomonas aeruginosa*, invasive *Aspergillus* and *Candida albicans* infections demonstrated high levels of CXCL8 and CCL2 in blood when compared to SARS patients without secondary infections, suggesting this profile may have potential utility as a biomarker of superinfection (Jiang et al., 2005). Together with IL-6, CXCL8 stimulation has been shown to reduce the capacity of activated DCs to induce T cell proliferation, potentially contributing to enhanced susceptibility towards secondary infections (Yoshikawa et al., 2009b).

Serum CXCL10 levels increase with SARS disease progression independently of secondary infection, and only wane during convalescence. Lymphopenia coincides with CXCL10 induction and also resolves during convalescence. Importantly, elevated serum CXCL10 was not strongly associated with non-SARS patients with atypical pneumonia (Jiang et al., 2005). *In vitro*, CXCL10 can be induced directly by either IFN-γ stimulation of monocytes or in SARS-CoV-infected epithelial cells. The induction and persistence of CXCL10 with progressive infection has suggested that it might serve as a biomarker for assessing the clinical stages of SARS infection (Jiang et al., 2005; Cameron et al., 2007).

#### Human Chemokine Gene Polymorphisms Associated With SARS

Human chemokine gene polymorphisms have been associated with SARS-CoV infectivity and pathogenicity. For example, the *CCL5* -28 CG/GG single nucleotide promoter polymorphism has been associated with increased risk of developing SARS and SARS mortality in a gene dosage-dependent manner (Ng et al., 2007; Lau and Peiris, 2009). *In vitro* studies demonstrated that the *CCL5* -28G allele enhances NF-κB binding, and increases promoter activity and CCL5 production in immune cells (Liu et al., 1999). Consistent with this, SARS presents in children as a mild disease and without elevations in plasma CCL5 levels (Hon et al., 2003; Ng et al., 2005). Nevertheless, the precise functional importance of CCL5 to SARS pathogenesis has not been defined. A functional single nucleotide polymorphism in *CCL2* which results in higher expression of the chemokine has also been reported to be an independent risk factor for increased susceptibility to SARS-CoV infection, but not mortality (Tu et al., 2015). Reported associations of *CCL2*, *CXCL9* and *CXCL10* polymorphisms with the incidence of infection and severity of SARS have been inconsistent (Ng et al., 2007; Lau and Peiris, 2009). Genome-Wide Association Studies using genetically diverse mouse strains have been performed to identify genetic loci contributing to SARS pathogenesis, however no genes or genetic networks relating to chemokine signaling were demonstrated to be directly involved (Gralinski et al., 2015).

#### Chemokines Associated With SARS Vaccine Responses

Numerous attenuated and inactivated SARS-CoV strains have been tested as vaccine candidates. Some have caused lung pathology, with infiltration of eosinophils, especially in aged mice. In particular, immunization with whole UV-inactivated SARS-CoV (UV-V) in mice provided partial protection towards subsequent SARS-CoV infection, with reduced weight loss and viral burden in the lung and enhanced survival, but induced pulmonary eosinophilia associated with increased expression in the lung of the eosinophil-targeted chemokine Ccl11. Toll-like receptor (TLR) stimulation as an adjuvant given with UV-V immunization inhibited Th2 skewing of the host immune response and reduced both Ccl11 levels and eosinophilic infiltration in lung. Instead, levels of the neutrophil-attracting chemokine Cxcl1 were higher in lung homogenates of challenged UV-V+TLR mice compared to UV-V mice without TLR stimulation. Transcriptomic studies of lung homogenates expanded on these observations, revealing that genes related to eosinophil chemotaxis were upregulated in UV-V mice but downregulated in TLR stimulated UV-V mice. Additionally, the genes associated with Th2 responses and chemotaxis and stimulation of eosinophils, Ccl17 and Ccl24, were upregulated in UV-V mice (Iwata-Yoshikawa et al., 2014).

Similar observations were found for a double-inactivated (UV irradiation and formalin) SARS-CoV strain adjuvanted with alum. Although this vaccine increased survival in aged mice challenged with a lethal and heterologous strain of SARS-CoV (S protein of a civet strain incorporated in the Urbani strain of SARS-CoV), severe lung pathology was observed, including perivascular and peribronchial cuffing and fibrinous exudates in alveolar parenchyma. Eosinophilia and heightened *Ccl11* expression in the lungs were also observed. *Cxcl1* was downregulated in the lungs of the challenged mock-vaccinated group compared to the vaccinated group (Bolles et al., 2011). SARS-CoV vaccines did not advance beyond Phase 1 studies in human.

#### The Functional Importance of Chemokines for SARS Immunopathogenesis

When mice genetically deficient for *Ccr1*, *Ccr2* or *Ccr5* were infected with SARS-CoV, greater weight loss and pulmonary damage were consistently observed. The pulmonary pathology was most severe in *Ccr1* knockout (KO) mice, and was associated with 40% mortality (Sheahan et al., 2008). Details regarding the nature of leukocyte infiltrates in these mice were not provided in this study.

When two Tg hACE2 mouse strains with varying levels of hACE2 expression, AC70 (higher hACE2, lethally susceptible to infection) and AC22 (lower hACE2, transient weight loss, not susceptible to lethality), were compared, induction of Cxcl1, Ccl2 and Ccl5 in the lung was delayed in the strain susceptible to lethal SARS-CoV infection compared to the resistant Tg mice. This delay was accompanied by reduced inflammatory infiltrates and a late resurgence of viral burden in lung prior to death (Yoshikawa et al., 2009a). Although mice deficient in Cxcl10 or its receptor Cxcr3 have not been tested specifically for SARS-CoV susceptibility, it is of interest that they demonstrate reduced disease severity and neutrophil content in BALF as well as enhanced survival in ARDS models involving intratracheal challenge with either PR8/H1N1 influenza virus or hydrochloric acid. It should be noted that the H1N1 viral burden in lung tissue of infected *Cxcl10* ^*−/−*^ and *Cxcr3* ^*−/−*^ mice were comparable to that of infected WT mice (Ichikawa et al., 2013).

### MERS-CoV

Like SARS-CoV, MERS-CoV most likely originated in bats but spillover to humans clearly involved passage through a different intermediate host, the dromedary camel (Azhar et al., 2014; Memish et al., 2014b; Cui et al., 2019). Since its emergence in Saudi Arabia in 2012, MERS has spread to 27 countries by human to human transmission linked to travel to or contacts with people from the Arabian Peninsula. The largest outbreak outside the Arabian Peninsula occurred in South Korea in 2015; only two cases have been reported in the United States, both in travelers from the Arabian Peninsula. The case fatality rate is ∼35%, by far the highest for human coronavirus infection and one of the highest mortality rates for any infectious disease of man (https://www.who.int/emergencies/mers-cov/en/, last accessed on August 17, 2020) (Zaki et al., 2012). Clinical manifestations range from asymptomatic or mild febrile illness to severe pneumonia, which may require mechanical ventilation and ICU admission. The major risk factors for mortality are similar to SARS: old age, male sex, smoking and the presence of comorbidities such as diabetes (Saad et al., 2014; Korea Centers for Diesase Control and Prevention, 2015; Garbati et al., 2016; Arabi et al., 2017b; Hui et al., 2018).

#### Receptor Usage and Lung Pathology

The majority of MERS patients with pneumonia show airspace and interstitial opacities on chest radiographs, which may be unilateral or bilateral as well as focal or diffuse in distribution (Min et al., 2016). Histopathological analysis reveals hemorrhagic pneumonia and exudative DAD, hyaline membrane formation and rare multinucleated syncytial cells (Ng et al., 2016; Alsaad et al., 2018). Similar pathological features have also been observed in MERS-CoV-infected mouse and nonhuman primate models (Table 3). In human lung autopsies, viral antigens were predominantly localized in type II pneumocytes and epithelial syncytial cells (Ng et al., 2016).

The functional receptor for MERS-CoV is dipeptidyl peptidase 4 (DPP4)/CD26, a type II transmembrane ectopeptidase. DPP4 interacts with the receptor binding domain of the S protein of MERS-CoV (Raj et al., 2013). Consistent with the expression of DPP4 on alveolar epithelium (Meyerholz et al., 2016), samples from the lower respiratory tract yielded the highest viral load (Memish et al., 2014a; Corman et al., 2016; Oh et al., 2016). Compared with patients who recovered or displayed mild symptoms, MERS-CoV viral load in the lower respiratory tract was greater and the viremia was more prolonged in patients who died or required oxygen supplementation (Min et al., 2016; Oh et al., 2016). Interestingly, DPP4 expression is enhanced on alveolar epithelium of smokers and patients with chronic lung diseases, including chronic obstructive pulmonary disease and cystic fibrosis (Meyerholz et al., 2016; Seys et al., 2018), which may explain in part why smoking and pre-existing lung disease are risk factors for MERS mortality (Korea Centers for Diesase Control and Prevention, 2015). Tg mice expressing human DPP4 (hDPP4) have facilitated the study of MERS-CoV infection. Viral pathogenesis has also been studied in rhesus macaques (de Wit et al., 2013) and common marmosets (Falzarano et al., 2014) (Table 3)*.*

#### Systemic Chemokine Upregulation

MERS-CoV infection induces systemic upregulation of inflammatory chemokines. CXCL10 and CCL2 levels in plasma are positively correlated with mortality (Min et al., 2016; Hong et al., 2018; Shin et al., 2019). In an anecdotal report consisting of two patients with different outcomes, the patient that succumbed to MERS-CoV infection had sustained CXCL10 levels in serum compared to the patient that recovered (Faure et al., 2014). In a second study, patients that required oxygen supplementation had higher neutrophil counts during the first week of hospitalization compared to patients who did not require supplemental oxygen. Moreover, the serum CXCL10 peak was higher and was delayed by a week compared to patients not requiring supplemental oxygen. Upregulation of CXCL10 also coincided with the peak of chest infiltrates and severity of pneumonia (Kim et al., 2016).

#### Chemokine Induction in Lungs and Lung Cell Lines

Studies investigating the effects of MERS-CoV infection on chemokine expression in lung are also limited. Neutrophil-targeted CXCL8 levels in the lower respiratory tract have been positively correlated with MERS-CoV viral load and mortality (Alosaimi et al., 2020). In addition, MERS-CoV induces higher mRNA and protein levels of CXCL8 compared to SARS-CoV in polarized airway epithelial Calu-3 cells (Lau et al., 2013). This may result in strong neutrophil recruitment in the lungs of infected patients, possibly contributing to the higher risk of severe disease and death in MERS patients compared to SARS patients.

MERS-CoV infection in patients is associated with CXCL10 expression in bronchial tissue (Chan et al., 2013). *In vitro* MERS-CoV can productively infect both MDMs and immature monocyte-derived dendritic cells (IMDDCs). While MERS-CoV was reported to continue to propagate in IMDDCs up to day 8 post infection, it was cleared from the supernatants of MDMs by day 6–8. The kinetics of CXCL10 induction differed in these cell types, with levels peaking faster and more sustainably in MDMs than IMDDCs, in which CXCL10 along with other chemokines and cytokines were very modestly expressed (Cong et al., 2018).

At the cellular level, compared to SARS-CoV, MERS-CoV induced lower amounts of CXCL10 in infected Calu-3 cells. In addition to lower induction of TNF-α and IFN-β in Calu-3 cells, lower CXCL10 levels in these cells may result in attenuated antiviral and pro-inflammatory responses against MERS-CoV, possibly contributing towards the higher production of MERS-CoV compared to SARS-CoV in these cells (Lau et al., 2013). Clearly, it will be important to test this experimentally to judge the suitability of CXCL10/CXCR3 signaling as a therapeutic target.

Type I IFN signaling in hematopoietic cells is strongly protective in MERS-CoV-infected mice (Channappanavar et al., 2019). In particular, human DPP4 (hDPP4)-knockin mice transplanted with *Ifnar* ^*−/−*^ bone marrow cells displayed greater mortality, as well as higher viral load and increased Cxcl1 expression in lung compared to hDPP4-knockin mice transplanted with syngeneic bone marrow cells. Since endogenous type I IFN expression in the lung peaked on day 2 after infection with a sublethal dose of a mouse-adapted strain of MERS-CoV in the model, the protective effect on mortality may depend on Type I IFN expression and function during an early time window after infection. Consistent with this, intranasal administration of recombinant IFN-β at 6 and 24 h post-infection protected mice against weight loss and death when compared to mice administered saline as well as mice administered recombinant IFN-β on days 2 and 4 post infection, which in turn had increased mortality compared to saline-treated controls. Mice given IFN-β in the protective time window also displayed reduced viral burden and Cxcl10 and Ccl2 gene expression in lung along with less infiltration of inflammatory monocytes/macrophages and neutrophils by day 4 post-infection, whereas these parameters were all increased in lungs of mice receiving IFN-β outside the protective time window. The time-sensitive effect of IFN-β administration on MERS pathogenesis in the model is similar to what was observed previously for SARS-CoV infection (Channappanavar et al., 2016). The pathogenic importance of Ccl2 induction in the MERS model was suggested by the ability of anti-Ccr2 antibody administration to deplete inflammatory monocytes/macrophages associated with significantly improved weight loss and survival rate of the recombinant IFN-β treated mice. To date there have been no studies selectively targeting chemokines or chemokine receptors, either genetically or pharmacologically in patients or animal models, to directly interrogate roles in pathogenesis.

#### MERS-CoV-Induced Chemokine Regulation

Different MERS-CoV strains can elicit different host responses. MERS-CoV SA 1 and MERS-CoV Eng 1, two genetically distinct MERS-CoV strains having 29 amino acid differences across the length of the viral genome and a deletion of 2 amino acids in the N protein of the MERS-CoV Eng 1 strain compared to the MERS-CoV SA 1 strain, were compared *in vitro*. Examination of innate host cell responses in Calu-3 cells demonstrated differential gene regulation, including for CCL5 and IFN-γ (Selinger et al., 2014). MERS-CoV-encoded proteins can directly modulate CXCL10. In particular, overexpression of MERS-CoV N protein upregulates CXCL10 in the human cell lines 293FT and A549 (Aboagye et al., 2018). Conversely, upon stimulation of the pattern recognition receptor MDA5, the MERS-CoV-encoded papain-like protease downregulated CCL5 and CXCL10 in HEK 293T cells (Mielech et al., 2014). This pathway may temporally regulate CXCL10 to delay and sustain its expression during MERS, potentially enhancing viral pathogenicity as evidenced by the positive association of MERS severity with circulating CXCL10 levels (Kim et al., 2016; Min et al., 2016; Hong et al., 2018; Shin et al., 2019). Mechanistically, the MERS-CoV accessory protein 4b interacts with the cellular importin karyopherin-α4, which normally binds to the NF-κB-p65 subunit and facilitates nuclear localization and activation of NF-κB, thereby resulting in cytoplasmic retention of NF-κB and attenuation of downstream inflammatory responses, such as chemokine induction (Canton et al., 2018).

#### Diabetes and CXCL10

A major risk factor for MERS mortality is diabetes (Assri et al., 2013; Korea Centers for Diesase Control and Prevention, 2015; Garbati et al., 2016; Arabi et al., 2017a; Habib et al., 2019). Diabetes induced by a high fat diet prolonged MERS-CoV disease, which included the duration and extent of body weight loss in male hDPP4 transgenic C57BL/6 mice compared to mice fed a normal diet. The lung viral burdens in the mouse groups were similar. The mechanism involved delayed onset and resolution of inflammation along with reduced infiltration of inflammatory monocytes/macrophages and CD4^+^ T cells in the lungs. Lung *Ccl2* expression coincided with the peak of inflammatory monocyte/macrophage accumulation in WT mice and was significantly higher during the initial phase of infection. During both phases of infection, *Ccl2* induction in diabetic mice was lower compared to WT mice. Cxcl10 expression in the lungs of diabetic mice was lower only during the initial stage of infection compared to non-diabetic infected mice (Kulcsar et al., 2019).

### SARS-CoV-2

SARS-CoV-2 was first reported and isolated in Wuhan, China in December 2019 from a disease now known as COVID-19 (Zhu et al., 2020). Koch’s postulates have been fulfilled establishing SARS-CoV-2 as the causative agent of COVID-19, which was declared a pandemic by the World Health Organization in March 2020. Sequence analysis strongly indicates that SARS-CoV-2 is most closely related to known bat coronaviruses (Boni et al., 2020); however, its evolutionary path to humans has not been defined, and remains controversial. Possibilities include direct spillover from a bat to humans, indirect spillover from a bat to humans through an intermediate host (as for SARS and MERS), and a leak from a laboratory studying bat coronaviruses, either from *bona fide* SARS-CoV-2 isolated from a natural sample or from a derivative of a natural virus generated by serial passage in laboratory animals and/or cell culture.

At present, there is no direct evidence that SARS-CoV-2 was present in any laboratory before it was first discovered in a patient, and detailed sequence analyses have been performed to argue that it most likely spilled over naturally (Andersen et al., 2020). The closest known related coronavirus sequence, designated RaTG13 (96.2% identical genome-wide to SARS-CoV-2), was identified by scientists from the Wuhan Institute of Virology (WIV) in a bat fecal sample isolated from a cave in Yunnan, China in 2013 (Zhou et al., 2020b). The complete sequence of RaTG13 was disclosed at the same time as the sequence of SARS-CoV-2 by the same WIV research group, which has been studying emerging infectious diseases and coronaviruses, particularly bat coronaviruses, for ∼20 years. The 3.8% nucleotide position differences separating RaTG13 and SARS-CoV-2 are estimated to represent decades in evolutionary distance in nature, although this gap could potentially be breached much more rapidly by serial passage in a laboratory, for which there is currently no direct evidence. Sequence analysis has suggested that any effort to derive SARS-CoV-2 by serial passage of RaTG13 or another related coronavirus would have had to occur *in vivo* since there is sequence evidence of conserved glycosylation sites in the S protein receptor binding domain indicating that it most likely evolved under immune pressure (Andersen et al., 2020).

Samples collected from the expedition in 2013 by WIV scientists to the bat caves in Yunnan province contained many other coronavirus sequences in addition to RaTG13 (Ge et al., 2016). Interestingly, this exploration was motivated by a recent outbreak of acute respiratory illness in 6 miners who had been working in a bat cave in Yunnan. The clinical histories of the 6 miners, 3 of whom did not survive their illness, were non-specific but in retrospect are clearly compatible with a diagnosis of severe COVID-19. The simplest explanation that links the Yunnan bat cave outbreak to the contemporaneous discovery of the RaTG13 sequence by Wuhan scientists in a Yunnan bat and the SARS-CoV-2 outbreak in Wuhan is that a bat coronavirus evolved the genetic features necessary to jump directly to humans, including the ability to efficiently use human ACE2 as a cell entry receptor and the acquisition of a furin cleavage site in the S protein that binds to ACE2 that is known to enhance ACE2 binding. Furin cleavage sites are unusual in viruses, but have been demonstrated previously to enhance cell entry, virulence and transmissibility in other viruses, including influenza. These cleavage sites can be acquired by serial passage in experimental animals, an example of so-called potential dual use gain-of-function research of concern which led to a moratarium on such research for almost a decade ending in 2017. Nevertheless, the cause of the miners’ illness was never established, and SARS-CoV-2 antibody titers have not been reported in these patients; moreover, it is unclear whether any patient samples still exist.

Since 7 years elapsed after the miners’ illnesses before COVID-19 was recognized, any causal relationship between the two events could have involved additional changes to the original virus to acquire more efficient person-to-person transmission in the general population outside of the intense environment of a bat cave. Importantly, the RaTG13 virus corresponding to the sequenced bat fecal sample collected by WIV scientists in 2013 was never isolated, nor has there been independent confirmation of its sequence, and unfortunately there is no more of the original sample remaining, separating the issue significantly from the realm of scientific questions that can be subjected to direct experimental scrutiny.

Extraordinary person-to-person viral transmissibility has established COVID-19 as a pandemic of historic proportions with extremely high total mortality despite a case fatality rate of less than 1%. Daily case incidence has risen and fallen over time in prominent waves that vary in number and shape in different countries and regions, as determined in large part by the stringency of the mitigation strategies governments deploy to modify social density and behavior and the degree of community compliance with them. The majority of COVID-19 patients are either asymptomatic or show mild symptoms that spontaneously resolve. After a median incubation period of ∼4 days, clinical manifestations commonly include fever, non-productive cough, myalgia, loss of the senses of smell and taste and dyspnea. Less common symptoms include fatigue, diarrhea, nausea and vomiting. A minority of patients rapidly progresses to ARDS and requires mechanical ventilation (Huang et al., 2020a; Chen et al., 2020; Guan et al., 2020; Wang et al., 2020). In addition to ARDS, multiorgan failure associated with intravascular coagulation is a major cause of COVID-19-related mortality (Zhou et al., 2020a). Multiple other presentations, disease courses and phenotypes have been described, including multifocal inflammatory syndrome in children (MIS-C), a Kawasaki’s Disease-like illness, and long hauler syndrome, a post COVID-19 chronic fatigue syndrome-like illness. Risk factors for COVID-19-related mortality include old age, male sex, neutrophilia, T cell lymphopenia, immunodeficiency, presence of secondary infections and comorbidities including diabetes, hypertension and diseases that affect the liver, kidney, cardiovascular, pulmonary and cerebrovascular systems (Chen et al., 2020; Huang et al., 2020a; Li et al., 2020; Ruan et al., 2020; Wang et al., 2020; Zhang et al., 2020; Zheng et al., 2020b; Zhou et al., 2020a)

#### Systemic Chemokine Upregulation

The severity of COVID-19 can be stratified based on plasma/serum chemokine levels. Serum monocyte-macrophage-directed CCL2, neutrophil-directed CXCL1 and CXCL8 and Th1 cell-directed CXCL9 levels are elevated in symptomatic, but not asymptomatic, infected individuals. Th1 cell-directed CXCL10 levels are upregulated in asymptomatic infected individuals compared to uninfected controls, and the levels are further elevated in symptomatic patients. Furthermore, CXCL10 levels are graded according to the severity of the disease (Laing et al., 2020). These inflammatory chemokines are all significantly reduced at the convalescent stage of the disease. Severe cases of COVID-19 also display higher levels of CCL7, CCL3 and CXCL9 compared to mild and moderate cases (Chi et al., 2020). Plasma levels of CXCL10, CCL2 and CCL3 were higher in ICU patients compared to non-ICU patients (Huang et al., 2020a). Also, the plasma CXCL10 levels were higher in non-stabilized patients compared to stabilized patients (Yale IMPACT research team et al., 2020). Furthermore, in a kinetic study, the plasma CXCL8, CCL2 and CCL7 levels were higher in fatal cases compared to the mild and/or severe cases of COVID-19. On the other hand, CCL5 levels were significantly lower in plasma of fatal cases compared to mild and severe cases. Plasma levels of the homeostatic chemokine CXCL12, neutrophil-targeted CXCL1, eosinophil-targeted CCL11 and the memory T cell-homing chemokine CCL27 remained high during infection and were not differentially regulated based on the severity of disease (Xu et al., 2020b). In another study, plasma CCL3 and CXCL10 levels were higher in COVID-19 patients with pneumonia and hypoxia requiring oxygen supplementation compared to patients without pneumonia and patients with pneumonia but not experiencing hypoxia (Young et al., 2020).

In severe cases, viral load has been reported to be higher in males, and more sustained in the elderly (Zheng et al., 2020a; Zheng et al., 2020b). Serum CCL3 and CCL7 levels were higher in male than female COVID-19 patients (Chi et al., 2020). In contrast, despite possessing comparable viral loads in saliva and nasopharyngeal swabs, male patients showed higher levels of plasma CXCL8 and CCL5 as well as a higher frequency of non-classical (CD14^low^CD16^+^) monocytes in the blood compared to female patients. Furthermore, plasma CCL5 levels were significantly higher and T cells were significantly lower in male patients possessing higher frequencies of circulating non-classical monocytes compared to male patients that possessed fewer non-classical monocytes. CCL5 levels were also higher in female patients that subsequently progressed to severe disease compared to the ones that stabilized (Yale impact research team et al., 2020).

Serum CCL17 levels have been suggested as a prognostic marker for severe cases of COVID-19. At an early phase of infection, patients with low CCL17 levels subsequently developed severe/critical COVID-19, whereas patients with comparatively higher CCL17 levels developed mild to moderate disease. Furthermore, CCL17 levels in common diseases (negative for SARS-CoV-2 RNA) including chronic hepatitis C, type 2 diabetes mellitus, chronic renal failure, chronic heart failure, interstitial pneumonia and rheumatoid arthritis were found to be higher than in COVID-19 patients at an early phase who went on to develop severe disease. The authors of this study also stated that CXCL9 and CXCL10 levels in sera surged and then suddenly dropped before the patients deteriorated and required oxygen support (Sugiyama et al., 2021). These markers along with the ones mentioned previously can perhaps be used as triage markers for predicting severe disease to prioritize for early therapeutic interventions. A Genome-wide Association Study in COVID-19 patients with respiratory failure identified a susceptibility locus at a chromosome 3p21.31 gene cluster, which includes the genes encoding the chemokine receptors CCR9, CXCR6 and XCR1, while genes for CCR1 and CCR2 flank the region (The Severe Covid-19 GWAS Group, 2020). Further studies are required to implicate individual host genes contributing towards COVID-19 severity in humans.

#### Animal Models and Receptor Usage

As mentioned previously, SARS-CoV-2, like SARS-CoV, binds to the human ACE2 receptor to gain entry into host cells (Wan et al., 2020). For host entry, the S protein must undergo activation by cleavage at two sites, S1/S2 and S2', which are mediated by host cellular proteases. The proprotein convertase, furin cleaves the S protein at the S1/S2 site, while TMPRSS2 cleaves at the S2' site. Cleavage at the S2' site is believed to trigger membrane fusion of the S protein (Bestle et al., 2020, Hoffmann et al., 2020). SARS-CoV-2 is unable to bind efficiently to mouse ACE2. Accordingly, neither WT mice nor mice deficient in RAG, STAT1 or type I and II IFN receptor signaling that do not express hACE2 are susceptible to SARS-CoV-2-induced weight loss and viral replication in the lung (Hassan et al., 2020). Therefore, mice have been engineered to be SARS-CoV-2 susceptible by transgenic introduction of hACE2 in the germline or by transiently transducing lung epithelium *in vivo* with replication-defective adeno-associated virus expressing hACE2 (Table 4). In addition to mice, intratracheal infections in rhesus macaques have demonstrated age-dependent development of pneumonia, with old monkeys developing more severe interstitial pneumonia than young monkeys. The presence of viral antigen was enhanced in an aged rhesus macaque compared to a younger monkey. The older macaque showed diffuse severe interstitial pneumonia with edema, extreme thickening of alveolar septum and abundant infiltration of inflammatory cells in the alveolar interstitium, whereas the development of these pathologies was milder in the younger macaque (Yu et al., 2020).

**Table 4:** Chemokine regulation in the lungs of animal models of SARS-CoV-2-induced viral pneumonia.

Animal model	Virus modification	Manifestations	Chemokine modulation in lungs	Reference
Mouse lung transfected with plasmid expressing hACE2	Wild-type	Inflammatory cell infiltration in lung parenchyma and interstitium; inflammatory cell aggregation in alveolar wall and spaces; Recruitment of neutrophils, monocytes, macrophages and T cells in BALF; No weight loss observed.	Upregulation of Ccl3, Ccl5, Cxcl1, Cxcl2 and Cxcl5	Liang et al., (2020)
*Cxcl5* ^*−/−*^ mice transfected with plasmid expressing hACE2	Wild-type	Reduced neutrophil recruitment in BALF of *Cxcl5* ^*−/−*^ mice along with less severe lung pathology and inflammatory cell aggregation in the bronchial walls and alveolar spaces compared to WT mice. Viral loads were comparable in both the mice groups	Ccl3 and Cxcl1 lower in Cxcl5^−/−^ mice compared to WT mice	Liang et al., (2020)
Mouse lung transduced with adenovirus 5 expressing hACE2	Wild-type	Interstitial pneumonia with perivascular inflammation and infiltration of numerous lymphocytes and macrophages; weight loss and mortality not observed; Presence of SARS-CoV-2 in pneumocytes in alveolar septa	Upregulation of Cxcl9 and Cxcl10	Han et al., (2020)
Mouse lung transduced with replication-deficient adenovirus 5 expressing hACE2	Wild-type	Increased vascular congestion, hemorrhage, perivascular and interstitial inflammatory cell infiltrates, necrotic cell debris and alveolar edema; Loss of body weight	Upregulation of Ccl2 and Cxcl10	Sun et al., (2020)
Tg male and female mice expressing hACE2 under the keratin 18 promoter	Wild-type	Viral antigen detected in pneumocytes and macrophages; Lung consolidation with inflammation and alveolar septal thickening with fibrin, edema and leukocyte infiltration; Vasculitis and weight loss.	Upregulation of Ccl2, Ccl5, Ccl9 and Cxcl10	Golden et al., (2020)
Tg male and female mice expressing hACE2 under the keratin 18 promoter	Wild-type	The majority of female mice survived upon infection with low dose viral challenge, while all male mice died	Male mice had higher Cxcl1 and Cxcl2 gene expression compared to female mice	Golden et al., (2020)
Mouse lung transiently transduced with replication-defective adenovirus 5 expressing hACE2	Wild-type	hACE2 transduced mice had virus infection in bronchiolar epithelial cells and pneumocytes; Neutrophil infiltration in perivascular and alveolar sites.	Upregulation of Cxcl10, Ccl2, Ccl5 and Cxcl11	Hassan et al., (2020)
Mouse lung transiently transduced with replication-defective adenovirus 5 expressing hACE2. These mice were also administered anti-IFNAR1 monoclonal antibodies	Wild-type	Infected IFNAR1 mAb treated mice showed enhanced weight loss, lung infiltration in perivascular and alveolar sites with macrophage accumulation. Neutralizing mAb against the SARS-CoV-2 receptor-binding domain ameliorated weight loss and virus burden in lungs of transduced, IFNAR1 mAb-treated mice Non-lethal infection with virus being undetectable by day 14 post infection.	The induction of these chemokines was reduced by neutralizing antibody specific to the SARS-CoV-2 S protein receptor-binding domain	Hassan et al., (2020)
Ferrets	Wild-type	Non-lethal infection with virus being undetectable by day 14 post infection.	Upregulation in expression of CCL8, CXCL9, CCL2, CCR5, CCR6, CXCR1, CXCR2 and CXCR7 in nasal washes.	Blanco-Melo et al., (2020)

#### Chemokine Regulation in Lung

Respiratory failure from ARDS is the leading cause of death in COVID-19 patients. The majority of patients, including many asymptomatic patients, develop diffuse bilateral pneumonia (Guan et al., 2020) with ground-glass opacity, which may progress to or co-exist with consolidation (Chen et al., 2020; Haseli et al., 2020; Huang et al., 2020a; Shi et al., 2020). Samples from the lower respiratory tract have a higher viral load than those from the upper respiratory tract (Huang et al., 2020b). Pathologic findings in the lung include bilateral DAD, interstitial thickening, pulmonary edema, proteinaceous exudates, hyaline membrane formation, presence of multinucleated syncytial cells, infiltration of T cells or inflammatory monocytes and evidence of widespread microthrombi (Ackermann et al., 2020; Barton et al., 2020; Martines et al., 2020; Tian et al., 2020; Xu et al., 2020a). Similar to SARS-CoV, SARS-CoV-2 is detectable in both type II pneumocytes and macrophages (Martines et al., 2020). The highly pro-inflammatory environment in the lungs of severe COVID-19 cases is reflected in the BALF, which contains high levels of pro-inflammatory MDMs, neutrophils and proliferating T cells compared to moderate cases. On the other hand, highly clonally expanded CD8^+^ T cells were more abundant in BALF from moderate cases compared to severe cases of COVID-19 (Liao et al., 2020). Analysis of normal lung tissue and tissue from post-mortem COVID-19 patients show two distinct immunopathological profiles. COVID-19 patients who died earlier after hospitalization had high local expression of interferon-stimulated genes (ISGs), cytokines and viral loads and limited pulmonary pathology. On the other hand, patients who died significantly later displayed lower ISG expression, low viral load, severe lung damage and abundant infiltration of activated CD8^+^ T cells and macrophages. Although intra-alveolar hemorrhage was not associated with the lung ISG status in these patients, it did positively correlate with median CXCL9/CXCL10/CXCL11 expression (Nienhold et al., 2020).

The levels of monocyte-, neutrophil- and Th1-specific chemokines are directly proportional to the severity of COVID-19 infection (Liao et al., 2020). CXCL8 was highly enriched in BALF from severe cases compared to healthy donors and mild cases of COVID-19, which correlated with abundant neutrophils in lungs of patients of that group (Park and Lee, 2020). Transcriptome studies on BALF cells from COVID-19 patients also reveal that inflammatory innate and Th1-associated chemokines, their receptors and their signaling pathways are highly upregulated (Gardinassi et al., 2020; Liao et al., 2020; Xiong et al., 2020; Zhou et al., 2020c). CCL3, CCL4, CCL5, XCL1 and XCL2 are highly expressed in virus-specific CD4^+^ T cells activated *in vitro* (Meckiff et al., 2020), suggesting that these cells may contribute to chemokine levels found in patient lung. At low multiplicity of infection, SARS-CoV-2 strongly induces various chemokines in normal human bronchial epithelial cells, whereas only low levels of IFN are expressed (Blanco-Melo et al., 2020). SARS-CoV-2 is much more sensitive to type I IFN pre-treatment than SARS-CoV *in vitro* (Lokugamage et al., 2020). While strongly inducing pro-inflammatory chemokines, suppression of IFN signaling likely allows for virus replication to occur, resulting in immunopathological damage to lungs (Blanco-Melo et al., 2020). *Ex vivo* studies with human lung tissue demonstrated that SARS-CoV and SARS-CoV-2 share similar cell tropism, with both viruses infecting type I and II pneumocytes and alveolar macrophages. Interestingly, SARS-CoV-2 was more efficient at replication than SARS-CoV, while gene expression of type I, II and III IFNs and pro-inflammatory cytokines including IL-1β, IL-6 and IL-12 were higher in SARS-CoV-infected cells. Furthermore, *CCL3*, *CXCL2*, *CXCL8* and *CXCL9* levels were also higher in SARS-CoV-infected cells, whereas the levels of *CXCL10* were higher in SARS-CoV-2 infected cells (Chu et al., 2020). These factors may contribute to higher transmissibility of SARS-CoV-2 through the naïve human population. Higher replication of SARS-COV-2 may be attributed to enhanced affinity of the virus’s receptor binding domain towards ACE2, when compared to that of SARS-CoV (Shang et al., 2020). Future studies will be needed to distinguish whether SARS-CoV is a superior inducer of the above mentioned pro-inflammatory factors or whether SARS-CoV-2 is more efficient at suppressing the innate and pro-inflammatory molecules or whether a combination of both mechanisms is at play.

RNA-sequencing studies on nasopharyngeal swabs of patients have revealed upregulation of the Th1 chemokines CXCL9, CXCL10, CXCL11 and CCL2 in patients with high viral load, while patients with lower viral loads had higher CXCL8 induction. Additionally, older patients showed reduced expression of CXCL9, CXCL10 and CXCL11 and their shared receptor CXCR3, suggesting potential impairment of trafficking/functioning of NK, cytotoxic T cells and other CXCR3-expressing cell types. *In silico* estimation of immune cell composition using gene expression data suggested a higher proportion of M1 macrophages, activated NK cells and DCs in high viral load samples, whereas naïve B and T cells, M2 macrophages and neutrophils were more abundant in lower viral load samples (Lieberman et al., 2020). Compared to lung biopsies from uninfected individuals, post-mortem lung samples from COVID-19 male patients older than 60 years demonstrated robust expression of CCL2, CCL8 and CCL11, with no IFN-I and IFN-III detection (Blanco-Melo et al., 2020). In throat swabs, viral shedding is sustained until death in fatal cases (Zhou et al., 2020a), which suggests a chronic pro-inflammatory viral-induced state in the absence of IFN induction, resulting in lung pathology and death. To date there are no published mechanistic studies aimed at precisely delineating the functional significance of chemokines upregulated by infection of animal models with SARS-CoV-2.

#### Therapeutic Considerations

Many COVID-19 vaccine candidates are in the pipeline with preliminary evidence of safety and immunologic efficacy at the level of inducing high titers of neutralizing antibodies. mRNA vaccines by Pfizer and Moderna have shown around 95% efficacy at preventing symptomatic SARS-CoV-2 infection. These vaccines have been approved by the FDA and their administration was initiated recently. Meanwhile, therapeutic options supported by evidence from randomized controlled trials are also beginning to emerge, with many clinical trials ongoing and being planned. Systemic and pulmonary chemokine levels may turn out to be useful prognostic markers to risk-stratify patients and accelerate interventions to reduce adverse outcomes. In addition, efforts need to advance to interrogate their relevance in disease pathogenesis and utility as therapeutic targets.

There are multiple clinical trials at various stages to assess chemokines for prediction and profiling of COVID-19 progression and severity (ClinicalTrials.gov Identifier: NCT04351711, NCT04365166, NCT04385108, NCT04423640, NCT04441502, NCT04474067). Also, various specific and non-specific inhibitors of chemokines are being tested to reduce the exaggerated pro-inflammatory response during COVID-19. The encouraging results of the Randomized Evaluation of COVID-19 Therapy trial advocate the low dose usage of the corticosteroid dexamethasone to reduce mortality in severe COVID-19 disease (The RECOVERY Collaborative Group, 2020). Upon dexamethasone treatment, dampening of systemic and lung chemokines by either increasing IL-10 or attenuating pro-inflammatory cytokines in these patients is likely, but requires future assessment. Targeted treatment in individuals with severe disease and poor prognosis through the use of chemokine signaling inhibitors is being evaluated. In particular, a phase 2 clinical trial is ongoing to test the efficacy of a human monoclonal antibody directed against IL-8, designated BMS-986253, in severe cases of COVID-19 (ClinicalTrials.gov Identifier: NCT04347226). The CCR5/CCR2b small molecule antagonist cenicriviroc reduces SARS-CoV-2 replication *in vitro* (Okamoto et al., 2020), and a phase 2 trial is testing the effectiveness of cenicriviroc to reduce the severity of COVID-19 (ClinicalTrials.gov Identifier: NCT04500418). Additionally, the CCR5-blocking antibody leronlimab has been reported to reduce plasma viremia in terminally ill COVID-19 patients (Patterson et al., 2020). Leronlimab was developed as an HIV entry inhibitor, acting by blocking CCR5 binding to HIV gp120 without blocking the binding of CCR5 ligands, a notable safety feature. However, this feature would not be predicted to have any efficacy in a disease like COVID-19 where the virus is not using CCR5 for entry and where the chemokines induced can act at CCR5 through an unblocked site, although it could potentially work by an as yet undefined alternative mechanism. Clinical trials enrolling mild-moderate (ClinicalTrials.gov Identifier: NCT04343651) and severe COVID-19 (ClinicalTrials.gov Identifier: NCT04347239) patients to assess the efficacy of leronlimab in ameliorating disease severity are ongoing. A phase 1 clinical trial to test the efficacy of maraviroc, an FDA-approved CCR5 antagonist in HIV/AIDS, is also in progress in moderate and severe cases of COVID-19 (ClinicalTrials.gov Identifier: NCT04435522). In addition to these clinical trials, it would be of significance to test the efficacy of CXCR3 antagonists in COVID-19 patients, considering that systemic CXCL10 levels are positively correlated with the severity of the disease (Huang et al., 2020a; Chi et al., 2020). Serum CX3CL1 levels are directly proportional to the severity of COVID-19 and decline during convalescence (Tong et al., 2020). A Phase 2 clinical trial to test the safety and efficacy of KAND567, a small molecule CX3CR1 blocker, in hospitalized COVID-19 patients is in progress (EudraCT Number: 2020-002322-85).

Furthermore, a clinical trial to examine the effects of the next-generation Bruton’s tyrosine kinase (BTK) inhibitor, acalabrutinib, on the immune response in COVID-19 patients is also underway (ClinicalTrials.gov Identifier: NCT04497948). Upon TLR stimulation in cells, BTKs upregulate chemokines via NF-κB activation. Hyperactivation of BTKs has been observed in blood monocytes of COVID-19 patients, and administration of acalabrutinib in an uncontrolled study involving a limited number of patients with severe COVID-19 appeared to considerably improve their clinical status (Roschewski et al., 2020).

A properly balanced innate immune response is required for initiation of the adaptive immune response and eventual viral clearance. However, coronaviruses modulate IFNs and chemokines to enable their replication prior to initiation of an exuberant chemokine response, leading to lung pathology and ARDS. Therefore, timely initiation to control the innate immune response may be necessary to clear the virus while causing minimum bystander tissue injury. Future studies with SARS-CoV-2 in mouse models may help elucidate the role of chemokines and their receptors in coronavirus infection. Mice deficient for chemokines and their receptors as well as the usage of specific chemokine receptor antagonists will aid in elucidating host-virus interactions. Furthermore, these studies will facilitate understanding of the role chemokines play in the poor outcomes experienced by many subjects with pre-existing comorbidities. These investigations will hopefully further the development of improved interventions to successfully reduce disease burden from SARS-CoV-2 as well as any future emergent coronaviruses.

## Summary

The COVID-19 pandemic emphasizes the need to study coronavirus-host responses, to define those that are beneficial and result in viral clearance and restoration of health, as well as those that become dysregulated and potentially contribute to ARDS, stroke, multisystem inflammatory syndrome in children/MIS-C and other severe life-threatening complications. Our focus on chemokine responses is motivated by the general importance of these molecules in coordinating immune responses and the tractability of GPCRs as targets for drug development.

**Figure 1:**
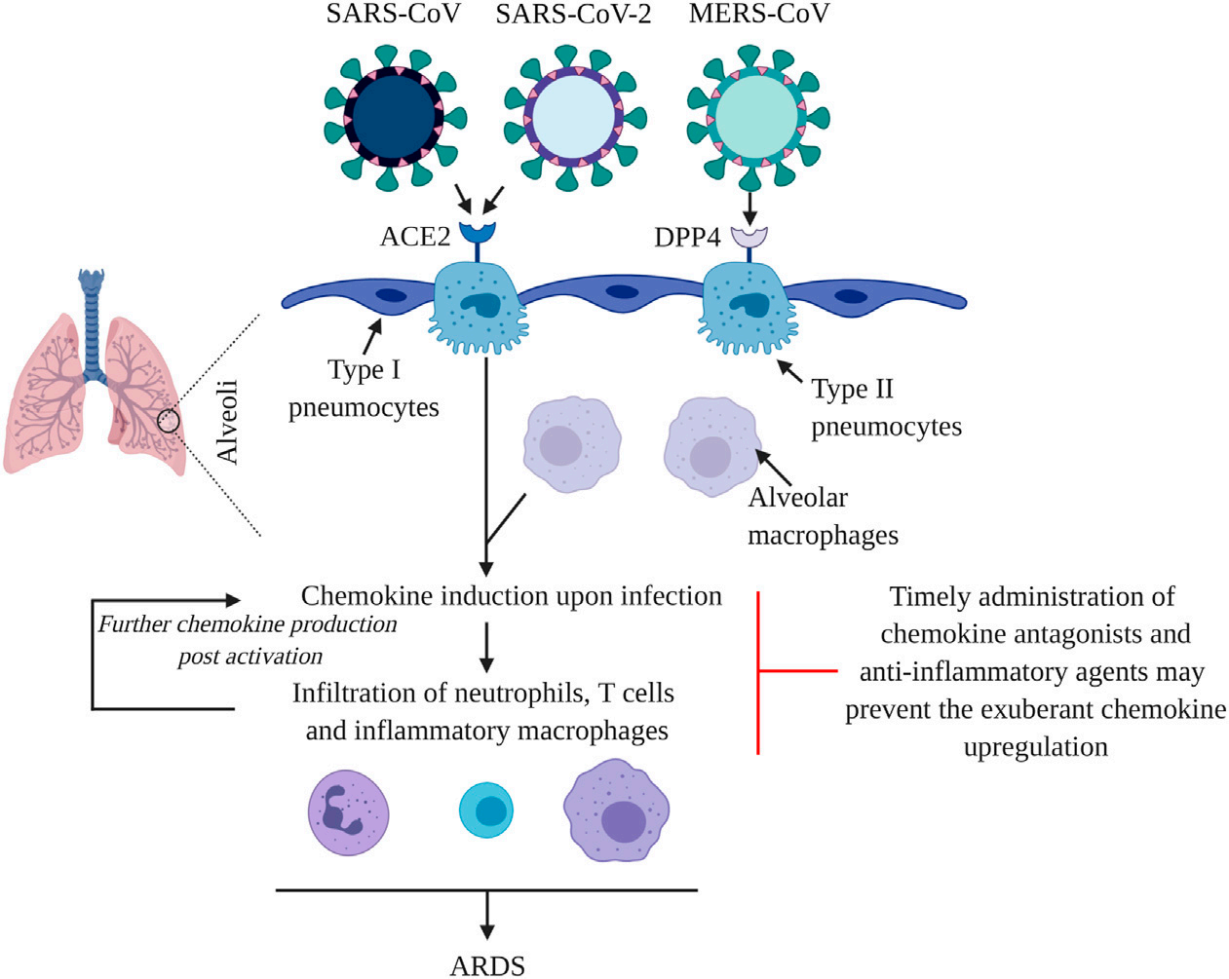
Virulent CoVs cause ARDS by inducing a chemokine/cytokine storm in lungs. SARS-CoV, SARS-CoV-2 and MERS-CoV primarily infect type II pneumocytes in the alveoli. The first two viruses use ACE2 as their receptor, while MERS-CoV uses DPP4 to gain entry into cells. The viruses propagate and subsequently infect other cell types including alveolar macrophages to induce chemokines, which mediate the infiltration of inflammatory macrophages, neutrophils and T cells. Upon activation, these cells further the production of chemokines, that contribute towards pulmonary damage and the development of ARDS. Systemic chemokine/cytokine storm may result in multiple organ dysfunction syndrome and fatality. Application of chemokine receptor antagonists and anti-inflammatory molecules such as corticosteroids and BTK inhibitors may dampen the leukocyte infiltration in lungs, reducing the ensuing morbidity and mortality.

Some features that distinguish SARS-CoV-2 from SARS-CoV and MERS-CoV are distinct receptor usage and affinity, intermediate host, transmissibility, proportion of asymptomatic patients and case fatality rate (Petrosillo et al., 2020) (Table 5). Uniquely, a prominent proportion of COVID-19 patients develop olfactory dysfunction including partial (hyposmia) or complete (anosmia) loss of smell (Meng et al., 2020). Furthermore, SARS-CoV-2 viral load peaks prior to the onset of symptoms, unlike infections with SARS-CoV and MERS-CoV, where their viral loads peak after onset of symptoms (Benefield et al., 2020). Despite these and other differences, the three epidemic coronaviruses may share a common pathway to induce ARDS in patients. They primarily infect alveolar epithelial cells to propagate and induce expression of pro-inflammatory molecules, including specific pro-inflammatory chemokines, to recruit and activate leukocytes within the lung. Subsequently, activated leukocytes may further enhance chemokine expression in a feed-forward manner, inducing an additional influx of leukocytes. This cascade may help drive development of ARDS, systemic upregulation of pro-inflammatory markers and multiple organ dysfunction syndrome, ultimately leading to death of the most severely affected patients (Figure 1). Although clinical observations and animal models of coronavirus infection have detailed the temporal and spatial distribution of chemokines in the infected lung, as we have reviewed, little has been done to precisely determine their functional roles and mechanisms in shaping outcome at the level of antiviral host defense and immunopathology. This major gap in knowledge identifies an opportunity for new research, with the hope of identifying new targets for therapeutic development. Fortunately, the chemokine field has developed critical gene-targeted mice and pharmacologic inhibitors that will allow these studies to progress systematically and with urgency given the threat imposed by COVID-19.

**Table 5:** Comparison of major features of the epidemic coronavirus.

	SARS-CoV	MERS-CoV	SARS-CoV-2
Disease	SARS	MERS	COVID-19
Duration	2003–2004	2012-present	2019-present
Country of origin	China	Saudi Arabia	China
Number of countries affected	29	27	189
Total cases	8,098	2,562	>44,312,806 (as of Oct 28, 2020)
Receptor	ACE2	DPP4	ACE2
TMPRSS2 dependence	Yes	Yes	Yes
Incubation period	2–7 days	2–14 days	2–14 days
Likely origin	Bats	Bats	Bats
Intermediate hosts	Palm civets	Camels	Unknown
Case fatality rate	10%	34.4%	<1% (estimate)
R_o_	2.0–3.0	0.9	1.8–3.6
Risk factors for mortality	Advanced age, male sex, presence of secondary infections, comorbidities like diabetes.	Advanced age, male sex, smoking, underlying diseases and comorbidities like diabetes.	Advanced age, male sex, immunodeficiency, presence of secondary infections, underlying diseases and comorbidities including diabetes and hypertension
Lung pathology in severe cases	DAD, consolidation, inflammatory cell infiltration, vascular thrombosis and presence of multinucleated giant cells	Hemorrhagic pneumonia, exudative DAD, inflammatory cell infiltration, vascular thrombosis and rare multinucleated syncytial cells	DAD, consolidation, hyaline membrane formation, inflammatory cell infiltration and presence of multinucleated giant cells
Systemic chemokine upregulation	Yes	Yes	Yes
Chemokines upregulated in lung	CCL2, CCL3, CCL27, CXCL2, CXCL8 and CXCL10	CXCL8	CCL2, CCL8, CCL11, CXCL8 and CXCL10

Data compiled from sources mentioned in the text and CDC and WHO resources.

## Author Contributions

PM conceived the idea of the review. SM and PM wrote and proofread the manuscript.

## Funding

This work was supported by funding from the Division of Intramural Research of the National Institute of Allergy and Infectious Diseases, NIH (project AI000615-28).

## Conflict of Interest

The authors declare that the research was conducted in the absence of any commercial or financial relationships that could be construed as a potential conflict of interest.

## Abbreviations

ACE2, Angiotensin-converting enzyme-2; ARDS, Acute respiratory distress syndrome; BALF, Bronchoalveolar lavage fluid; BTK, Bruton’s tyrosine kinase; CAP, Community-acquired pneumonia; CoV, Coronavirus; DAD, Diffuse alveolar damage; DCs, Dendritic cells; DPP4, Dipeptidyl peptidase 4; E, Envelope; GPCRs, G protein-coupled receptors; hACE2, Human angiotensin-converting enzyme-2; hDPP4, Human dipeptidyl peptidase 4; ICU, Intensive care unit; IFN, Interferon; IFNAR, IFNαβ receptor; IMDDCs, Immature monocyte-derived dendritic cells; ISGs, Interferon stimulated genes; KC, Neutrophil-related chemokine KC; KO, Knockout; M, Membrane; MA, Mouse-adapted; MDM, Monocyte-derived macrophages; MERS-CoV, Middle East respiratory syndrome coronavirus; MIS-C, Multifocal inflammatory syndrome in children; N, Nucleocapsid; NSP, Nonstructural protein; S, Spike; SARS-CoV, Severe acute respiratory syndrome coronavirus; Tg, Transgenic; TLR, Toll-like receptor; TRMPRSS2, Transmembrane protease serine type 2; UV-V, UV-inactivated SARS-CoV; WIV, Wuhan institute of virology; WT, Wild type.

## References

[B1] Aboagye J. O. Yew C. W. Ng O. W. Monteil V. M. Mirazimi A. Tan Y. J. (2018). Overexpression of the nucleocapsid protein of Middle East respiratory syndrome coronavirus up-regulates CXCL10. Biosci. Rep. 38, BSR20181059 10.1042/BSR20181059 30242057 PMC6200698

[B2] Ackermann M. Verleden S. E. Kuehnel M. Haverich A. Welte T. Laenger F. (2020). Pulmonary vascular endothelialitis, thrombosis, and angiogenesis in covid-19. N. Engl. J. Med. 383, 120–128. 10.1056/NEJMoa2015432 32437596 PMC7412750

[B3] Agrawal A. S. Garron T. Tao X. Peng B. H. Wakamiya M. Chan T. S. (2015). Generation of a transgenic mouse model of Middle East respiratory syndrome coronavirus infection and disease. J. Virol. 89, 3659–3670. 10.1128/JVI.03427-14 25589660 PMC4403411

[B4] Alosaimi B. Hamed M. E. Naeem A. Alsharef A. A. AlQahtani S. Y. AlDosari K. M. (2020). MERS-CoV infection is associated with downregulation of genes encoding Th1 and Th2 cytokines/chemokines and elevated inflammatory innate immune response in the lower respiratory tract. Cytokine. 126, 154895 10.1016/j.cyto.2019.154895 31706200 PMC7128721

[B5] Alssad K. O. Hajeer A. H. Al Balwi M. Al Moaiqel M. Al Oudah N. Al Ajlan A. (2018). Histopathology of Middle East respiratory syndrome coronovirus (MERS-CoV) infection - clinicopathological and ultrastructural study. Histopathology. 72, 516–524. 10.1111/his.13379 28858401 PMC7165512

[B6] Andersen K. G. Rambaut A. Lipkin W. I. Holmes E. C. Garry R. F. (2020). The proximal origin of SARS-CoV-2. Nat. Med. 26, 450–452. 10.1038/s41591-020-0820-9 32284615 PMC7095063

[B7] Arabi Y. M. Al-Omari A. Mandourah Y. Al-Hameed F. Sindi A. A. Alraddadi B. (2017a). Critically ill patients with the Middle East respiratory syndrome: a multicenter retrospective cohort study. Crit. Care Med. 45, 1683–1695. 10.1097/CCM.0000000000002621 28787295

[B8] Arabi Y. M. Balkhy H. H. Hayden F. G. Bouchama A. Luke T. Baillie J. K. (2017b). Middle East respiratory syndrome. N. Engl. J. Med. 376, 584–594. 10.1056/NEJMsr1408795 28177862 PMC5362064

[B9] Assri A. McGeer A. Perl T. M. Price C. S. Al Rabeeah A. A. Cummings D. A. (2013). Hospital outbreak of Middle East respiratory syndrome coronavirus. N. Engl. J. Med. 369, 407–416. 10.1056/NEJMoa1306742 23782161 PMC4029105

[B10] Azhar E. I. El-Kafrawy S. A. Farraj S. A. Hassan A. M. Al-Saeed M. S. Hashem A. M. (2014). Evidence for camel-to-human transmission of MERS coronavirus. N. Engl. J. Med. 370, 2499–2505. 10.1056/NEJMoa1401505 24896817

[B11] Barton L. M. Duval E. J. Stroberg E. Ghosh S. Mukhopadhyay S. (2020). COVID-19 autopsies, Oklahoma, USA. Am. J. Clin. Pathol. 153, 725–733. 10.1093/ajcp/aqaa062 32275742 PMC7184436

[B12] Beigel J. H. Tomashek K. M. Dodd L. E. Mehta A. K. Zingman B. S. Kalil A. C. (2020). Remdesivir for the treatment of covid-19 — preliminary report. N. Engl. J. Med. 383, 1813–1826. 10.1056/NEJMoa2007764 32445440 PMC7262788

[B13] Benefield A. E. Skrip L. A. Clement A. Althouse R. A. Chang S. Althouse B. M. (2020). SARS-CoV-2 viral load peaks prior to symptom onset: a systematic review and individual-pooled analysis of coronavirus viral load from 66 studies. Infectious Diseases (except HIV/AIDS). [Epub ahead of print]. 10.1101/2020.09.28.20202028

[B200] Bestle D. Heindl M. R. Limburg H. Van Lam van T. Pilgram O. Moulton H. (2020). TMPRSS2 and furin are both essential for proteolytic activation of SARS-CoV-2 in human airway cells. Life Sci. Alliance. 3, e202000786 10.26508/lsa.202000786 32703818 PMC7383062

[B14] Blanco-Melo D. Nilsson-Payant B. E. Liu W. C. Uhl S. Hoagland D. Møller R. (2020). Imbalanced host response to SARS-CoV-2 drives development of COVID-19. Cell. 181, 1036–1045.e9. 10.1016/j.cell.2020.04.026 32416070 PMC7227586

[B15] Bolles M. Deming D. Long K. Agnihothram S. Whitmore A. Ferris M. (2011). A double-inactivated severe acute respiratory syndrome coronavirus vaccine provides incomplete protection in mice and induces increased eosinophilic proinflammatory pulmonary response upon challenge. J. Virol. 85, 12201–12215. 10.1128/JVI.06048-11 21937658 PMC3209347

[B16] Booth C. M. Matukas L. M. Tomlinson G. A. Rachlis A. R. Rose D. B. Dwosh H. A. (2003). Clinical features and short-term outcomes of 144 patients with SARS in the greater toronto area. J. Am. Med. Assoc. 289, 2801 10.1001/jama.289.21.JOC30885 12734147

[B17] Boni M. F. Lemey P. Jiang X. Lam T. T.-Y. Perry B. W. Castoe T. A. (2020). Evolutionary origins of the SARS-CoV-2 sarbecovirus lineage responsible for the COVID-19 pandemic. Nat Microbiol. 50, 1408–1417. 10.1038/s41564-020-0771-4 32724171

[B18] Cameron M. J. Ran L. Xu L. Danesh A. Bermejo-Martin J. F. Cameron C. M. (2007). Interferon-mediated immunopathological events are associated with atypical innate and adaptive immune responses in patients with severe acute respiratory syndrome. J. Virol. 81, 8692–8706. 10.1128/JVI.00527-07 17537853 PMC1951379

[B19] Cameron M. J. Kelvin A. A. Leon A. J. Cameron C. M. Ran L. Xu L. (2012). Lack of innate interferon responses during SARS coronavirus infection in a vaccination and reinfection ferret model. PLoS ONE. 7, e45842 10.1371/journal.pone.0045842 23029269 PMC3454321

[B20] Canton J. Fehr A. R. Fernandez-Delgado R. Gutierrez-Alvarez F. J. Sanchez-Aparicio M. T. García-Sastre A. (2018). MERS-CoV 4b protein interferes with the NF-κB-dependent innate immune response during infection. PLoS Pathog. 14, e1006838 10.1371/journal.ppat.1006838 29370303 PMC5800688

[B21] Castelli V. Cimini A. Ferri C. (2020). Cytokine storm in COVID-19: "when you come out of the storm, you won't Be the same person who walked in".” Front. Immunol. 11, 2132 10.3389/fimmu.2020.02132 32983172 PMC7492381

[B22] Chan R. W. Chan M. C. Agnihothram S. Chan L. L. Kuok D. I. Fong J. H. (2013). Tropism of and innate immune responses to the novel human betacoronavirus lineage C virus in human ex vivo respiratory organ cultures. J. Virol. 87, 6604–6614. 10.1128/JVI.00009-13 23552422 PMC3676115

[B23] Chang Y. J. Liu C. Y. Chiang B. L. Chao Y. C. Chen C. C. (2004). Induction of IL-8 release in lung cells via activator protein-1 by recombinant baculovirus displaying severe acute respiratory syndrome-coronavirus Spike proteins: identification of two functional regions. J. Immunol. 173, 7602–7614. 10.4049/jimmunol.173.12.7602 15585888

[B24] Channappanavar R. Fehr A. R. Vijay R. Mack M. Zhao J. Meyerholz D. K. (2016). Dysregulated type I interferon and inflammatory monocyte-macrophage responses cause lethal pneumonia in SARS-CoV-infected mice. Cell Host Microbe. 19, 181–193. 10.1016/j.chom.2016.01.007 26867177 PMC4752723

[B25] Channappanavar R. Fett C. Mack M. Ten Eyck P. P. Meyerholz D. K. Perlman S. (2017). Sex-based differences in susceptibility to severe acute respiratory syndrome coronavirus infection. J. Immunol. 198, 4046–4053. 10.4049/jimmunol.1601896 28373583 PMC5450662

[B26] Channappanavar R. Fehr A. R. Zheng J. Wohlford-Lenane C. Abrahante J. E. Mack M. (2019). IFN-I response timing relative to virus replication determines MERS coronavirus infection outcomes. J. Clin. Invest. 129, 3625–3639. 10.1172/JCI126363 31355779 PMC6715373

[B27] Chen C. Y. Lee C. H. Liu C. Y. Wang J. H. Wang L. M. Perng R. P. (2005). Clinical features and outcomes of severe acute respiratory syndrome and predictive factors for acute respiratory distress syndrome. J. Chin. Med. Assoc. 68, 4–10. 10.1016/S1726-4901(09)70124-8 15742856 PMC7129615

[B28] Chen I. Y. Chang S. C. Wu H. Y. Yu T. C. Wei W. C. Lin S. (2010a). Upregulation of the chemokine (C-C motif) ligand 2 via a severe acute respiratory syndrome coronavirus spike-ACE2 signaling pathway. J. Virol. 84, 7703–7712. 10.1128/JVI.02560-09 20484496 PMC2897593

[B29] Chen J. Lau Y. F. Lamirande E. W. Paddock C. D. Bartlett J. H. Zaki S. R. (2010b). Cellular immune responses to severe acute respiratory syndrome coronavirus (SARS-CoV) infection in senescent BALB/c mice: CD4+ T cells are important in control of SARS-CoV infection. J. Virol. 84, 1289–1301. 10.1128/JVI.01281-09 19906920 PMC2812346

[B30] Chen N. Zhou M. Dong X. Qu J. Gong F. Han Y. (2020). Epidemiological and clinical characteristics of 99 cases of 2019 novel coronavirus pneumonia in Wuhan, China: a descriptive study. Lancet. 395, 507–513. 10.1016/S0140-6736(20)30211-7 32007143 PMC7135076

[B31] Cheung C. Y. Poon L. L. Ng I. H. Luk W. Sia S. F. Wu M. H. (2005). Cytokine responses in severe acute respiratory syndrome coronavirus-infected macrophages in vitro: possible relevance to pathogenesis. J. Virol. 79, 7819–7826. 10.1128/JVI.79.12.7819-7826.2005 15919935 PMC1143636

[B32] Chi Y. Ge Y. Wu B. Zhang W. Wu T. Wen T. (2020). Serum cytokine and chemokine profile in relation to the severity of coronavirus disease 2019 in China. J. Infect. Dis. 222, 746–754. 10.1093/infdis/jiaa363 32563194 PMC7337752

[B33] Chien J. Y. Hsueh P. R. Cheng W. C. Yu C. J. Yang P. C. (2006). Temporal changes in cytokine/chemokine profiles and pulmonary involvement in severe acute respiratory syndrome. Respirology. 11, 715–722. 10.1111/j.1440-1843.2006.00942.x 17052299 PMC7192207

[B34] Choi K. W. Chau T. N. Tsang O. Tso E. Chiu M. C. Tong W. L. (2003). Outcomes and prognostic factors in 267 patients with severe acute respiratory syndrome in Hong Kong. Ann. Intern. Med. 139, 715 10.7326/0003-4819-139-9-200311040-00005 14597455

[B35] Chu H. Chan J. F. Wang Y. Yuen T. T. Chai Y. Hou Y. (2020). Comparative replication and immune activation profiles of SARS-CoV-2 and SARS-CoV in human lungs: an ex vivo study with implications for the pathogenesis of COVID-19. Clin. Infect. Dis. 71, 1400–1409. 10.1093/cid/ciaa410 32270184 PMC7184390

[B36] Cong Y. Hart B. J. Gross R. Zhou H. Frieman M. Bollinger L. (2018). MERS-CoV pathogenesis and antiviral efficacy of licensed drugs in human monocyte-derived antigen-presenting cells. PLoS ONE. 13, e0194868 10.1371/journal.pone.0194868 29566060 PMC5864050

[B37] Coleman C. M. Sisk J. M. Halasz G. Zhong J. Beck S. E. Matthews K. L. (2017). CD8+ T cells and macrophages regulate pathogenesis in a mouse model of Middle East respiratory syndrome. J. Virol. 91, e01825-16 10.1128/JVI.01825-16 27795435 PMC5165197

[B38] Coperchini F. Chiovato L. Croce L. Magri F. Rotondi M. (2020). The cytokine storm in COVID-19: an overview of the involvement of the chemokine/chemokine-receptor system. Cytokine Growth Factor Rev. 53, 25–32. 10.1016/j.cytogfr.2020.05.003 32446778 PMC7211650

[B39] Corman V. M. Albarrak A. M. Omrani A. S. Albarrak M. M. Farah M. E. Almasri M. (2016). Viral shedding and antibody response in 37 patients with Middle East respiratory syndrome coronavirus infection. Clin. Infect. Dis. 62, 477–483. 10.1093/cid/civ951 26565003 PMC7108065

[B40] Coronaviridae Study Group of the International Committee on Taxonomy of Viruses (2020). The species Severe acute respiratory syndrome-related coronavirus: classifying 2019-nCoV and naming it SARS-CoV-2. Nat Microbiol. 5, 536–544. 10.1038/s41564-020-0695-z 32123347 PMC7095448

[B41] Cui J. Li F. Shi Z. L. (2019). Origin and evolution of pathogenic coronaviruses. Nat. Rev. Microbiol. 17, 181–192. 10.1038/s41579-018-0118-9 30531947 PMC7097006

[B42] Danesh A. Seneviratne C. Cameron C. M. Banner D. Devries M. E. Kelvin A. A. (2008). Cloning, expression and characterization of ferret CXCL10. Mol. Immunol. 45, 1288–1297. 10.1016/j.molimm.2007.09.018 18006061 PMC5653245

[B43] Day C. W. Baric R. Cai S. X. Frieman M. Kumaki Y. Morrey J. D. (2009). A new mouse-adapted strain of SARS-CoV as a lethal model for evaluating antiviral agents *in vitro* and *in vivo* . Virology. 395, 210–222. 10.1016/j.virol.2009.09.023 19853271 PMC2787736

[B44] DeDiego M. L. Nieto-Torres J. L. Regla-Nava J. A. Jimenez-Guardeño J. M. Fernandez-Delgado R. Fett C. (2014). Inhibition of NF-κB-mediated inflammation in severe acute respiratory syndrome coronavirus-infected mice increases survival. J. Virol. 88, 913–924. 10.1128/JVI.02576-13 24198408 PMC3911641

[B45] de Lang A. Baas T. Teal T. Leijten L. M. Rain B. Osterhaus A. D. (2007). Functional genomics highlights differential induction of antiviral pathways in the lungs of SARS-CoV-infected macaques. PLoS Pathog. 3, e112 10.1371/journal.ppat.0030112 17696609 PMC1941749

[B46] de Wit E. Rasmussen A. L. Falzarano D. Bushmaker T. Feldmann F. Brining D. L. (2013). Middle East respiratory syndrome coronavirus (MERS-CoV) causes transient lower respiratory tract infection in rhesus macaques. Proc. Natl. Acad. Sci. U.S.A. 110, 16598–16603. 10.1073/pnas.1310744110 24062443 PMC3799368

[B47] Falzarano D. de Wit E. Rasmussen A. L. Feldmann F. Okumura A. Scott D. P. (2013). Treatment with interferon-α2b and ribavirin improves outcome in MERS-CoV-infected rhesus macaques. Nat. Med. 19, 1313–1317. 10.1038/nm.3362 24013700 PMC4093902

[B48] Falzarano D. de Wit E. Feldmann F. Rasmussen A. L. Okumura A. Peng X. (2014). Infection with MERS-CoV causes lethal pneumonia in the common marmoset. PLoS Pathog. 10, e1004250 10.1371/journal.ppat.1004250 25144235 PMC4140844

[B49] Faure E. Poissy J. Goffard A. Fournier C. Kipnis E. Titecat M. (2014). Distinct immune response in two MERS-CoV-infected patients: can we go from bench to bedside? PLoS ONE. 9, e88716 10.1371/journal.pone.0088716 24551142 PMC3925152

[B50] Frieman M. B. Chen J. Morrison T. E. Whitmore A. Funkhouser W. Ward J. M. (2010). SARS-CoV pathogenesis is regulated by a STAT1 dependent but a type I, II and III interferon receptor independent mechanism. PLoS Pathog. 6, e1000849 10.1371/journal.ppat.1000849 20386712 PMC2851658

[B51] Franks T. J. Chong P. Y. Chui P. Galvin J. R. Lourens R. M. Reid A. H. (2003). Lung pathology of severe acute respiratory syndrome (SARS): a study of 8 autopsy cases from Singapore. Hum. Pathol. 34, 743–748. 10.1016/S0046-8177(03)00367-8 14506633 PMC7119137

[B52] Garbati M. A. Fagbo S. F. Fang V. J. Skakni L. Joseph M. Wani T. A. (2016). A comparative study of clinical presentation and risk factors for adverse outcome in patients hospitalised with acute respiratory disease due to MERS coronavirus or other causes. PLoS ONE. 11, e0165978 10.1371/journal.pone.0165978 27812197 PMC5094725

[B53] Gardinassi L. G. Souza C. O. S. Sales-Campos H. Fonseca S. G. (2020). Immune and metabolic signatures of COVID-19 revealed by transcriptomics data reuse. Front. Immunol. 11, 1636 10.3389/fimmu.2020.01636 32670298 PMC7332781

[B54] Ge X. Y. Wang N. Zhang W. Hu B. Li B. Zhang Y. Z. (2016). Coexistence of multiple coronaviruses in several bat colonies in an abandoned mineshaft. Virol. Sin. 31, 31–40. 10.1007/s12250-016-3713-9 26920708 PMC7090819

[B55] Glass W. G. Rosenberg H. F. Murphy P. M. (2003). Chemokine regulation of inflammation during acute viral infection. Curr. Opin. Allergy Clin. Immunol. 3, 467–473. 10.1097/00130832-200312000-00008 14612671

[B56] Glass W. G. Subbarao K. Murphy B. Murphy P. M. (2004). Mechanisms of host defense following severe acute respiratory syndrome-coronavirus (SARS-CoV) pulmonary infection of mice. J. Immunol. 173, 4030–4039. 10.4049/jimmunol.173.6.4030 15356152

[B57] Golden J. W. Cline C. R. Zeng X. Garrison A. R. Carey B. D. Mucker E. M. (2020). Human angiotensin-converting enzyme 2 transgenic mice infected with SARS-CoV-2 develop severe and fatal respiratory disease. JCI Insight. 5, e142032 10.1172/jci.insight.142032 PMC7566707 32841215

[B58] Gu J. Gong E. Zhang B. Zheng J. Gao Z. Zhong Y. (2005). Multiple organ infection and the pathogenesis of SARS. J. Exp. Med. 202, 415–424. 10.1084/jem.20050828 16043521 PMC2213088

[B59] Gralinski L. E. Ferris M. T. Aylor D. L. Whitmore A. C. Green R. Frieman M. B. (2015). Genome wide identification of SARS-CoV susceptibility loci using the collaborative cross. PLoS Genet. 11, e1005504 10.1371/journal.pgen.1005504 26452100 PMC4599853

[B60] Griffith J. W. Sokol C. L. Luster A. D. (2014). Chemokines and chemokine receptors: positioning cells for host defense and immunity. Annu. Rev. Immunol. 32, 659–702. 10.1146/annurev-immunol-032713-120145 24655300

[B61] Guan W. Ni Z. Hu Y. Liang W. Ou C. He J. (2020). Clinical characteristics of coronavirus disease 2019 in China. N. Engl. J. Med. 382, 1708–1720. 10.1056/NEJMoa2002032 32109013 PMC7092819

[B62] Habib A. M. G. Ali M. A. E. Zouaoui B. R. Taha M. A. H. Mohammed B. S. Saquib N. (2019). Clinical outcomes among hospital patients with Middle East respiratory syndrome coronavirus (MERS-CoV) infection. BMC Infect. Dis. 19, 870 10.1186/s12879-019-4555-5 31640578 PMC6805532

[B63] Han K. Blair R. V. Iwanaga N. Liu F. Russell-Lodrigue K. E. Qin Z. (2020). Lung expression of human ACE2 sensitizes the mouse to SARS-CoV-2 infection. Am J Respir Cell Mol Biol. [Epub ahead of print]. 10.1165/rcmb.2020-0354OC PMC7781002 32991819

[B64] Haseli S. Khalili N. Bakhshayeshkaram M. Sanei Taheri M. Moharramzad Y. (2020). Lobar distribution of COVID-19 pneumonia based on chest computed tomography findings; A retrospective study. Arch Acad Emerg Med. 8, e55. 32440666 PMC7212068

[B65] Hassan A. O. Case J. B. Winkler E. S. Thackray L. B. Kafai N. M. Bailey A. L. (2020). A SARS-CoV-2 infection model in mice demonstrates protection by neutralizing antibodies. Cell. 182, 744–753.e4. 10.1016/j.cell.2020.06.011 32553273 PMC7284254

[B66] He L. Ding Y. Zhang Q. Che X. He Y. Shen H. (2006). Expression of elevated levels of pro-inflammatory cytokines in SARS-CoV-infected ACE2+ cells in SARS patients: relation to the acute lung injury and pathogenesis of SARS. J. Pathol. 210, 288–297. 10.1002/path.2067 17031779 PMC7167655

[B67] Hoffmann M. Kleine-Weber H. Schroeder S. Krüger N. Herrler T. Erichsen S. (2020). SARS-CoV-2 cell entry depends on ACE2 and TMPRSS2 and is blocked by a clinically proven protease inhibitor. Cell. 181, 271–280.e8. 10.1016/j.cell.2020.02.052 32142651 PMC7102627

[B68] Hong K. H. Choi J. P. Hong S. H. Lee J. Kwon J. S. Kim S. M. (2018). Predictors of mortality in Middle East respiratory syndrome (MERS). Thorax. 73, 286–289. 10.1136/thoraxjnl-2016-209313 28724637

[B69] Hu B. Zeng L. P. Yang X. L. Ge X. Y. Zhang W. Li B. (2017). Discovery of a rich gene pool of bat SARS-related coronaviruses provides new insights into the origin of SARS coronavirus. PLoS Pathog. 13, e1006698 10.1371/journal.ppat.1006698 29190287 PMC5708621

[B70] Huang K. J. Su I. J. Theron M. Wu Y. C. Lai S. K. Liu C. C. (2005). An interferon-gamma-related cytokine storm in SARS patients. J. Med. Virol. 75, 185–194. 10.1002/jmv.20255 15602737 PMC7166886

[B71] Huang C. Wang Y. Li X. Ren L. Zhao J. Hu Y. (2020a). Clinical features of patients infected with 2019 novel coronavirus in Wuhan, China. Lancet. 395, 497–506. 10.1016/S0140-6736(20)30183-5 31986264 PMC7159299

[B72] Huang Y. Chen S. Yang Z. Guan W. Liu D. Lin Z. (2020b). SARS-CoV-2 viral load in clinical samples from critically ill patients. Am. J. Respir. Crit. Care Med. 201, 1435–1438. 10.1164/rccm.202003-0572LE 32293905 PMC7258645

[B73] Hon K. Leung C. Cheng W. Chan P. Chu W. Kwan Y. (2003). Clinical presentations and outcome of severe acute respiratory syndrome in children. Lancet. 361, 1701–1703. 10.1016/S0140-6736(03)13364-8 12767737 PMC7112484

[B74] Hsueh P. R. Chen P. J. Hsiao C. H. Yeh S. H. Cheng W. C. Wang J. L. (2004). Patient data, early SARS epidemic, taiwan. Emerging Infect Dis. 10, 489–493. 10.3201/eid1003.030571 15109419

[B75] Hui D. S. Azhar E. I. Kim Y. J. Memish Z. A. Oh M. Zumla A. (2018). Middle East respiratory syndrome coronavirus: risk factors and determinants of primary, household, and nosocomial transmission. Lancet Infect. Dis. 18, e217–e227. 10.1016/S1473-3099(18)30127-0 29680581 PMC7164784

[B76] Ichikawa A. Kuba K. Morita M. Chida S. Tezuka H. Hara H. (2013). CXCL10-CXCR3 enhances the development of neutrophil-mediated fulminant lung injury of viral and nonviral origin. Am. J. Respir. Crit. Care Med. 187, 65–77. 10.1164/rccm.201203-0508OC 23144331 PMC3927876

[B77] Iwata-Yoshikawa N. Uda A. Suzuki T. Tsunetsugu-Yokota Y. Sato Y. Morikawa S. (2014). Effects of toll-like receptor stimulation on eosinophilic infiltration in lungs of BALB/c mice immunized with UV-inactivated severe acute respiratory syndrome-related coronavirus vaccine. J. Virol. 88, 8597–8614. 10.1128/JVI.00983-14 24850731 PMC4135953

[B78] Iwata-Yoshikawa N. Okamura T. Shimizu Y. Hasegawa H. Takeda M. Nagata N. (2019a). TMPRSS2 contributes to virus spread and immunopathology in the airways of murine models after coronavirus infection. J. Virol. 93, e01815–e01818. 10.1128/JVI.01815-18 30626688 PMC6401451

[B79] Iwata-Yoshikawa N. Okamura T. Shimizu Y. Kotani O. Sato H. Sekimukai H. (2019b). Acute respiratory infection in human dipeptidyl peptidase 4-transgenic mice infected with Middle East respiratory syndrome coronavirus. J. Virol. 93, e01818 10.1128/JVI.01818-18 30626685 PMC6401458

[B80] Jiang Y. Xu J. Zhou C Wu Z. Zhong S. Liu J. (2005). Characterization of cytokine/chemokine profiles of severe acute respiratory syndrome. Am. J. Respir. Crit. Care Med. 171, 850–857. 10.1164/rccm.200407-857OC 15657466

[B81] Jimenez-Guardeño J. M. Nieto-Torres J. L. DeDiego M. L. Regla-Nava J. A. Fernandez-Delgado R. Castaño-Rodriguez C. (2014). The PDZ-binding motif of severe acute respiratory syndrome coronavirus envelope protein is a determinant of viral pathogenesis. PLoS Pathog. 10, e1004320 10.1371/journal.ppat.1004320 25122212 PMC4133396

[B82] Kim E. S. Choe P. G. Park W. B. Oh H. S. Kim E. J. Nam E. Y. (2016). Clinical progression and cytokine profiles of Middle East respiratory syndrome coronavirus infection. J Korean Med Sci. 31, 1717 10.3346/jkms.2016.31.11.1717 27709848 PMC5056202

[B83] Korea Centers for Disease Control and Prevention (2015). Middle East Respiratory Syndrome Coronavirus Outbreak in the Republic of Korea, 2015. Osong Public Health and Research Perspectives. 6, 269–278. 10.1016/j.phrp.2015.08.006 26473095 PMC4588443

[B84] Kong S. L. Chui P. Lim B. Salto-Tellez M. (2009). Elucidating the molecular physiopathology of acute respiratory distress syndrome in severe acute respiratory syndrome patients. Virus Res. 145, 260–269. 10.1016/j.virusres.2009.07.014 19635508 PMC7114434

[B85] Ksiazek T. G. Erdman D. Goldsmith C. S. Zaki S. R. Peret T. Emery S. (2003). A novel coronavirus associated with severe acute respiratory syndrome. N. Engl. J. Med. 348, 1953–1966. 10.1056/NEJMoa030781 12690092

[B86] Kulcsar K. A. Coleman C. M. Beck S. E. Frieman M. B. (2019). Comorbid diabetes results in immune dysregulation and enhanced disease severity following MERS-CoV infection. JCI Insight. 4, e131774 10.1172/jci.insight.131774 PMC6824443 31550243

[B87] Kumaki Y. Day C. W. Bailey K. W. Wandersee M. K. Wong M. H. Madsen J. R. (2010). Induction of interferon-gamma-inducible protein 10 by SARS-CoV infection, interferon alfacon 1 and interferon inducer in human bronchial epithelial Calu-3 cells and BALB/c mice. Antivir. Chem. Chemother. 20, 169–177. 10.3851/IMP1477 20231782

[B88] Kuri T. Zhang X. Habjan M. Martínez-Sobrido L. García-Sastre A. Yuan Z. (2009). Interferon priming enables cells to partially overturn the SARS coronavirus-induced block in innate immune activation. J. Gen. Virol. 90, 2686–2694. 10.1099/vir.0.013599-0 19625461 PMC2888313

[B89] Laing A. G. Lorenc A. del Molino del Barrio I. Das A. Fish M. Monin L. (2020). A dynamic COVID-19 immune signature includes associations with poor prognosis. Nat. Med. 26, 1623–1635. 10.1038/s41591-020-1038-6 32807934

[B90] Lau S. K. Lau C. C. Chan K. H. Li C. P. Chen H. Jin D. Y. (2013). Delayed induction of proinflammatory cytokines and suppression of innate antiviral response by the novel Middle East respiratory syndrome coronavirus: implications for pathogenesis and treatment. J. Gen. Virol. 94, 2679–2690. 10.1099/vir.0.055533-0 24077366

[B91] Lau Y. L. Peiris J. S. (2009). Association of cytokine and chemokine gene polymorphisms with severe acute respiratory syndrome. Hong Kong Med. J. 15 (Suppl 2), 43–46. 19258635

[B92] Law A. H. Lee D. C. Cheung B. K. Yim H. C. Lau A. S. (2007). Role for nonstructural protein 1 of severe acute respiratory syndrome coronavirus in chemokine dysregulation. J. Virol. 81, 416–422. 10.1128/JVI.02336-05 17035307 PMC1797241

[B93] Law H. K. Cheung C. Y. Ng H. Y. Sia S. F. Chan Y. O. Luk W. (2005). Chemokine up-regulation in SARS-coronavirus-infected, monocyte-derived human dendritic cells. Blood. 106, 2366–2374. 10.1182/blood-2004-10-4166 15860669 PMC1895271

[B94] Law H. K. Cheung C. Sia S. Chan Y. Peiris J. S. Lau Y. (2009). Toll-like receptors, chemokine receptors and death receptor ligands responses in SARS coronavirus infected human monocyte derived dendritic cells. BMC Immunol. 10, 35 10.1186/1471-2172-10-35 19505311 PMC2700820

[B95] Lee N. Hui D. Wu A. Chan P. Cameron P. Joynt G. M. (2003). A major outbreak of severe acute respiratory syndrome in Hong Kong. N. Engl. J. Med. 348, 1986–1994. 10.1056/NEJMoa030685 12682352

[B96] Li K. Wohlford-Lenane C. Perlman S. Zhao J. Jewell A. K. Reznikov L. R. (2016). Middle East respiratory syndrome coronavirus causes multiple organ damage and lethal disease in mice transgenic for human dipeptidyl peptidase 4. J. Infect. Dis. 213, 712–722. 10.1093/infdis/jiv499 26486634 PMC4747621

[B97] Li K. Wohlford-Lenane C. L. Channappanavar R. Park J. E. Earnest J. T. Bair T. B. (2017). Mouse-adapted MERS coronavirus causes lethal lung disease in human DPP4 knockin mice. Proc. Natl. Acad. Sci. U.S.A. 114, E3119–E3128. 10.1073/pnas.1619109114 28348219 PMC5393213

[B98] Li X. Xu S. Yu M. Wang K. Tao Y. Zhou Y. (2020). Risk factors for severity and mortality in adult COVID-19 inpatients in Wuhan. J. Allergy Clin. Immunol. 146, 110–118. 10.1016/j.jaci.2020.04.006 32294485 PMC7152876

[B99] Li W. Moore M. J. Vasilieva N. Sui J. Wong S. K. Berne M. A. (2003). Angiotensin-converting enzyme 2 is a functional receptor for the SARS coronavirus. Nature. 426, 450–454. 10.1038/nature02145 14647384 PMC7095016

[B100] Liang Y. Li H. Li J. Yang Z.-N. Li J.-L. Zheng H.-W. (2020). Role of neutrophil chemoattractant CXCL5 in SARS-CoV-2 infection-induced lung inflammatory innate immune response in an *in-vivo* hACE2 transfection mouse model. Zool. Res. 41, 1–11. 10.24272/j.issn.2095-8137.2020.118 33045777 PMC7671918

[B101] Liao M. Liu Y. Yuan J. Wen Y. Xu G. Zhao J. (2020). Single-cell landscape of bronchoalveolar immune cells in patients with COVID-19. Nat. Med. 26, 842–844. 10.1038/s41591-020-0901-9 32398875

[B102] Lieberman N. A. P. Peddu V. Xie H. Shrestha L. Huang M.-L. Mears M. C. (2020). *In vivo* antiviral host response to SARS-CoV-2 by viral load, sex, and age. PLoS Biol. 18, e3000849 10.1371/journal.pbio.3000849 32898168 PMC7478592

[B103] Liu H. Chao D. Nakayama E. E. Taguchi H. Goto M. Xin X. (1999). Polymorphism in RANTES chemokine promoter affects HIV-1 disease progression. Proc. Natl. Acad. Sci. U.S.A. 96, 4581–4585. 10.1073/pnas.96.8.4581 10200305 PMC16375

[B104] Lokugamage K. G. Hage A. de Vries M. Valero-Jimenez A. M. Schindewolf C. Dittmann M. (2020). Type I interferon susceptibility distinguishes SARS-CoV-2 from SARS-CoV. Virol 94, e01410-20, /jvi/94/23/JVI.01410-20.atom. 10.1128/JVI.01410-20 PMC7654262 32938761

[B105] Martines R. B. Ritter J. M. Matkovic E. Gary J. Bollweg B. C. Bullock H. (2020). Pathology and pathogenesis of SARS-CoV-2 associated with fatal coronavirus disease, United States. Emerg. Infect. Dis. 26, 2005 10.3201/eid2609.202095 32437316 PMC7454055

[B106] Meckiff B. J. Ramírez-Suástegui C. Fajardo V. Chee S. J. Kusnadi A. Simon H. (2020). Single-cell transcriptomic analysis of SARS-CoV-2 reactive CD4 ^+^ T cells. Immunology. 10.1101/2020.06.12.148916 PMC7534589 33096020

[B107] Matthay M. A. Zemans R. L. Zimmerman G. A. Arabi Y. M. Beitler J. R. Mercat A. (2019). Acute respiratory distress syndrome. Nat Rev Dis Primers. 5, 18 10.1038/s41572-019-0069-0 30872586 PMC6709677

[B108] McCray P. B. Pewe L. Wohlford-Lenane C. Hickey M. Manzel L. Shi L. (2007). Lethal infection of K18-hACE2 mice infected with severe acute respiratory syndrome coronavirus. J. Virol. 81, 813–821. 10.1128/JVI.02012-06 17079315 PMC1797474

[B109] Memish Z. A. Al-Tawfiq J. A. Makhdoom H. Q. Assiri A. Alhakeem R. F. Albarrak A. (2014a). Respiratory tract samples, viral load, and genome fraction yield in patients with Middle East respiratory syndrome. J. Infect. Dis. 210, 1590–1594. 10.1093/infdis/jiu292 24837403 PMC7107391

[B110] Memish Z. A. Cotten M. Meyer B. Watson S. J. Alsahafi A. J. Al Rabeeah A. A. (2014b). Human infection with MERS coronavirus after exposure to infected camels, Saudi Arabia, 2013. Emerging Infect Dis. 20, 1012–1015. 10.3201/eid2006.140402 PMC4036761 24857749

[B111] Meng X. Deng Y. Dai Z. Meng Z. (2020). COVID-19 and anosmia: a review based on up-to-date knowledge. Am. J. Otolaryngol. 41, 102581 10.1016/j.amjoto.2020.102581 32563019 PMC7265845

[B112] Meyerholz D. K. Lambertz A. M. McCray P. B. (2016). Dipeptidyl peptidase 4 distribution in the human respiratory tract: implications for the Middle East respiratory syndrome. Am. J. Pathol. 186, 78–86. 10.1016/j.ajpath.2015.09.014 26597880 PMC4715219

[B113] Mielech A. M. Kilianski A. Baez-Santos Y. M. Mesecar A. D. Baker S. C. (2014). MERS-CoV papain-like protease has deISGylating and deubiquitinating activities. Virology. 450–451, 64–70. 10.1016/j.virol.2013.11.040 PMC3957432 24503068

[B114] Min C. K. Cheon S. Ha N. Y. Sohn K. M. Kim Y. Aigerim A. (2016). Comparative and kinetic analysis of viral shedding and immunological responses in MERS patients representing a broad spectrum of disease severity. Sci. Rep. 6, 25359 10.1038/srep25359 27146253 PMC4857172

[B115] Mossel E. C. Wang J. Jeffers S. Edeen K. E. Wang S. Cosgrove G. P. (2008). SARS-CoV replicates in primary human alveolar type II cell cultures but not in type I-like cells. Virology. 372, 127–135. 10.1016/j.virol.2007.09.045 18022664 PMC2312501

[B116] Nagata N. Iwata N. Hasegawa H. Fukushi S. Harashima A. Sato Y. (2008). Mouse-passaged severe acute respiratory syndrome-associated coronavirus leads to lethal pulmonary edema and diffuse alveolar damage in adult but not young mice. Am. J. Pathol. 172, 1625–1637. 10.2353/ajpath.2008.071060 18467696 PMC2408422

[B117] Ng D. L. Al Hosani F. Keating M. K. Gerber S. I. Jones T. L. Metcalfe M. G. (2016). Clinicopathologic, immunohistochemical, and ultrastructural findings of a fatal case of Middle East respiratory syndrome coronavirus infection in the United Arab Emirates, april 2014. Am. J. Pathol. 186, 652–658. 10.1016/j.ajpath.2015.10.024 26857507 PMC7093852

[B118] Ng P. C. Lam K. C. W. Li A. M. Wong C. K. Leung T. F. Cheng F. W. T. (2005). Chemokine response in children with SARS. Arch. Dis. Child. 90, 422–423. 10.1136/adc.2004.053660 15781938 PMC1720352

[B119] Ng M. W. Zhou G. Chong W. P. Lee L. W. Law H. K. Zhang H. (2007). The association of RANTES polymorphism with severe acute respiratory syndrome in Hong Kong and Beijing Chinese. BMC Infect. Dis. 7, 50 10.1186/1471-2334-7-50 17540042 PMC1899505

[B120] Nicholls J. M. Butany J. Poon L. L. Chan K. H. Beh S. L. Poutanen S. (2006). Time course and cellular localization of SARS-CoV nucleoprotein and RNA in lungs from fatal cases of SARS. PLoS Med. 3, e27 10.1371/journal.pmed.0030027 16379499 PMC1324951

[B121] Nicholls J. M. Poon L. L. Lee K. C. Ng W. F. Lai S. T. Leung C. Y. (2003). Lung pathology of fatal severe acute respiratory syndrome. Lancet. 361, 1773–1778. 10.1016/S0140-6736(03)13413-7 12781536 PMC7112492

[B122] Nienhold R. Ciani Y. Koelzer V. H. Tzankov A. Haslbauer J. D. Menter T. (2020). Two distinct immunopathological profiles in autopsy lungs of COVID-19. Nat. Commun. 11, 5086 10.1038/s41467-020-18854-2 33033248 PMC7546638

[B123] Oh M. Park W. B. Choe P. G. Choi S. J. Kim J. I. Chae J. (2016). Viral load kinetics of MERS coronavirus infection. N. Engl. J. Med. 375, 1303–1305. 10.1056/NEJMc1511695 27682053

[B124] Okamoto M. Toyama M. Baba M. (2020). The chemokine receptor antagonist cenicriviroc inhibits the replication of SARS-CoV-2 *in vitro* . Antiviral Res. 182, 104902 10.1016/j.antiviral.2020.104902 32739404 PMC7392080

[B125] Patterson B. K. Seethamraju H. Dhody K. Corley M. J. Kazempour K. Lalezari J. (2021). CCR5 inhibition in critical COVID-19 patients decreases inflammatory cytokines, increases CD8 T-cells, and decreases SARS-CoV2 RNA in plasma by day 14. Int. J. Infect. Disease. 103, 25–32. 10.1016/j.ijid.2020.10.101 33186704 PMC7654230

[B126] Park J. H. Lee H. K. (2020). Re-analysis of single cell transcriptome reveals that the NR3C1-CXCL8-neutrophil Axis determines the severity of COVID-19. Front. Immunol. 11, 2145 10.3389/fimmu.2020.02145 32983174 PMC7485000

[B127] Peiris J. Chu C. Cheng V. Chan K. Hung I. Poon L. (2003a). Clinical progression and viral load in a community outbreak of coronavirus-associated SARS pneumonia: a prospective study. Lancet. 361, 1767–1772. 10.1016/S0140-6736(03)13412-5 12781535 PMC7112410

[B128] Peiris J. Lai S. Poon L. Guan Y. Yam L. Lim W. (2003b). Coronavirus as a possible cause of severe acute respiratory syndrome. Lancet. 361, 1319–1325. 10.1016/S0140-6736(03)13077-2 12711465 PMC7112372

[B129] Petersen E. Koopmans M. Go U. Hamer D. H. Petrosillo N. Castelli F. (2020a). Comparing SARS-CoV-2 with SARS-CoV and influenza pandemics. Lancet Infect. Dis. 10.1016/S1473-3099(20)30484-9 PMC7333991 32628905

[B130] Petersen E. Koopmans M. Go U. Hamer D. H. Petrosillo N. Castelli F. (2020b). Comparing SARS-CoV-2 with SARS-CoV and influenza pandemics. Lancet Infect. Dis. 20, e238–e244. 10.1016/S1473-3099(20)30484-9 32628905 PMC7333991

[B131] Petrosillo N. Viceconte G. Ergonul O. Ippolito G. Petersen E. (2020). COVID-19, SARS and MERS: are they closely related? Clin. Microbiol. Infect. 26, 729–734. 10.1016/j.cmi.2020.03.026 32234451 PMC7176926

[B132] Qian Z. Travanty E. A. Oko L. Edeen K. Berglund A. Wang J. (2013). Innate immune response of human alveolar type II cells infected with severe acute respiratory syndrome-coronavirus. Am. J. Respir. Cell Mol. Biol. 48, 742–748. 10.1165/rcmb.2012-0339OC 23418343 PMC3727876

[B133] Raj V. S. Mou H. Smits S. L. Dekkers D. H. Müller M. A. Dijkman R. (2013). Dipeptidyl peptidase 4 is a functional receptor for the emerging human coronavirus-EMC. Nature. 495, 251–254. 10.1038/nature12005 23486063 PMC7095326

[B134] Rockx B. Baas T. Zornetzer G. A. Haagmans B. Sheahan T. Frieman M. (2009). Early upregulation of acute respiratory distress syndrome-associated cytokines promotes lethal disease in an aged-mouse model of severe acute respiratory syndrome coronavirus infection. J. Virol. 83, 7062–7074. 10.1128/JVI.00127-09 19420084 PMC2704758

[B135] Roschewski M. Lionakis M. S. Sharman J. P. Roswarski J. Goy A. Monticelli M. A. (2020). Inhibition of Bruton tyrosine kinase in patients with severe COVID-19. Sci. Immunol. 5, eabd0110 10.1126/sciimmunol.abd0110 32503877 PMC7274761

[B136] Ruan Q. Yang K. Wang W. Jiang L. Song J. (2020). Clinical predictors of mortality due to COVID-19 based on an analysis of data of 150 patients from Wuhan, China. Intensive Care Med. 46, 846–848. 10.1007/s00134-020-05991-x 32125452 PMC7080116

[B137] Saad M. Omrani A. S. Baig K. Bahloul A. Elzein F. Matin M. A. (2014). Clinical aspects and outcomes of 70 patients with Middle East respiratory syndrome coronavirus infection: a single-center experience in Saudi Arabia. Int. J. Infect. Dis. 29, 301–306. 10.1016/j.ijid.2014.09.003 25303830 PMC7110769

[B138] Selinger C. Tisoncik-Go J. Menachery V. D. Agnihothram S. Law G. Chang J. (2014). Cytokine systems approach demonstrates differences in innate and pro-inflammatory host responses between genetically distinct MERS-CoV isolates. BMC Genom. 15, 1161 10.1186/1471-2164-15-1161 PMC4522970 25534508

[B139] Seys L. J. M. Widagdo W. Verhamme F. M. Kleinjan A. Janssens W. Joos G. F. (2018). DPP4, the Middle East respiratory syndrome coronavirus receptor, is upregulated in lungs of smokers and chronic obstructive pulmonary disease patients. Clin. Infect. Dis. 66, 45–53. 10.1093/cid/cix741 29020176 PMC7108100

[B140] Shang J. Ye G. Shi K. Wan Y. Luo C. Aihara H. (2020). Structural basis of receptor recognition by SARS-CoV-2. Nature. 581, 221–224. 10.1038/s41586-020-2179-y 32225175 PMC7328981

[B141] Sheahan T. Morrison T. E. Funkhouser W. Uematsu S. Akira S. Baric R. S. (2008). MyD88 is required for protection from lethal infection with a mouse-adapted SARS-CoV. PLoS Pathog. 4, e1000240 10.1371/journal.ppat.1000240 19079579 PMC2587915

[B142] Shi H. Han X. Jiang N. Cao Y. Alwalid O. Gu J. (2020). Radiological findings from 81 patients with COVID-19 pneumonia in Wuhan, China: a descriptive study. Lancet Infect. Dis. 20, 425–434. 10.1016/S1473-3099(20)30086-4 32105637 PMC7159053

[B143] Shin H. S. Kim Y. Kim G. Lee J. Y. Jeong I. Joh J. S. (2019). Immune responses to Middle East respiratory syndrome coronavirus during the acute and convalescent phases of human infection. Clin. Infect. Dis. 68, 984–992. 10.1093/cid/ciy595 30060038 PMC7108191

[B144] Smits S. L. de Lang A. van den Brand J. M. Leijten L. M. van IJcken W. F. Eijkemans M. J. (2010). Exacerbated innate host response to SARS-CoV in aged non-human primates. PLoS Pathog. 6, e1000756 10.1371/journal.ppat.1000756 20140198 PMC2816697

[B145] Smits S. L. van den Brand J. M. de Lang A. Leijten L. M. van IJcken W. F. van Amerongen G. (2011). Distinct severe acute respiratory syndrome coronavirus-induced acute lung injury pathways in two different nonhuman primate species. J. Virol. 85, 4234–4245. 10.1128/JVI.02395-10 21325418 PMC3126247

[B146] Spellberg B. (2008). Dr. William H. Stewart: mistaken or maligned? Clin. Infect. Dis. 47, 294 10.1086/589579 18564938

[B147] Sugiyama M. Kinoshita N. Ide S. Nomoto H. Nakamoto T. Saito S. (2021). Serum CCL17 level becomes a predictive marker to distinguish between mild/moderate and severe/critical disease in patients with COVID-19. Gene. 766, 145145 10.1016/j.gene.2020.145145 32941953 PMC7489253

[B148] Sun J. Zhuang Z. Zheng J. Li K. Wong R. L. Liu D. (2020). Generation of a broadly useful model for COVID-19 pathogenesis, vaccination, and treatment. Cell. 182, 734–743.e5. 10.1016/j.cell.2020.06.010 32643603 PMC7284240

[B149] Tang N. L. Chan P. K. Wong C. K. To K. F. Wu A. K. Sung Y. M. (2005). Early enhanced expression of interferon-inducible protein-10 (CXCL-10) and other chemokines predicts adverse outcome in severe acute respiratory syndrome. Clin. Chem. 51, 2333–2340. 10.1373/clinchem.2005.054460 16195357 PMC7108146

[B150] The RECOVERY Collaborative Group (2020). Dexamethasone in hospitalized patients with covid-19 — preliminary report. N. Engl. J. Med. [Epub ahead of print]. 10.1056/NEJMoa2021436 PMC7383595 32678530

[B151] The Severe Covid-19 GWAS Group (2020). Genomewide association study of severe covid-19 with respiratory failure. N. Engl. J. Med. 383, 1522–1534. 10.1056/NEJMoa2020283 32558485 PMC7315890

[B152] Tian S. Hu W. Niu L. Liu H. Xu H. Xiao S. Y. (2020). Pulmonary pathology of early-phase 2019 novel coronavirus (COVID-19) pneumonia in two patients with lung cancer. J. Thorac. Oncol. 15, 700–704. 10.1016/j.jtho.2020.02.010 32114094 PMC7128866

[B153] Tong M. Jiang Y. Xia D. Xiong Y. Zheng Q. Chen F. (2020). Elevated expression of serum endothelial cell adhesion molecules in COVID-19 patients. J. Infect. Dis. 222, 894–898. 10.1093/infdis/jiaa349 32582936 PMC7337874

[B154] Tsang K. W. Ho P. L. Ooi G. C. Yee W. K. Wang T. Chan-Yeung M. (2003). A cluster of cases of severe acute respiratory syndrome in Hong Kong. N. Engl. J. Med. 348, 1977–1985. 10.1056/NEJMoa030666 12671062

[B155] Tse G. M. To K. F. Chan P. K. S. Lo A. W. I. Ng K. C. Wu A. (2004). Pulmonary pathological features in coronavirus associated severe acute respiratory syndrome (SARS). J. Clin. Pathol. 57, 260–265. 10.1136/jcp.2003.013276 14990596 PMC1770245

[B156] Tseng C. T. Huang C. Newman P. Wang N. Narayanan K. Watts D. M. (2007). Severe acute respiratory syndrome coronavirus infection of mice transgenic for the human angiotensin-converting enzyme 2 virus receptor. J. Virol. 81, 1162–1173. 10.1128/JVI.01702-06 17108019 PMC1797529

[B157] Tu X. Chong W. P. Zhai Y. Zhang H. Zhang F. Wang S. (2015). Functional polymorphisms of the CCL2 and MBL genes cumulatively increase susceptibility to severe acute respiratory syndrome coronavirus infection. J. Infect. 71, 101–109. 10.1016/j.jinf.2015.03.006 25818534 PMC7112636

[B158] Xiong Y. Liu Y. Cao L. Wang D. Guo M. Jiang A. (2020). Transcriptomic characteristics of bronchoalveolar lavage fluid and peripheral blood mononuclear cells in COVID-19 patients. Emerg. Microb. Infect. 9, 761–770. 10.1080/22221751.2020.1747363 PMC7170362 32228226

[B159] Xu Z. Shi L. Wang Y. Zhang J. Huang L. Zhang C. (2020a). Pathological findings of COVID-19 associated with acute respiratory distress syndrome. Lancet Respir Med. 8, 420–422. 10.1016/S2213-2600(20)30076-X 32085846 PMC7164771

[B160] Xu Z.-S. Shu T. Kang L. Wu D. Zhou X. Liao B.-W. (2020b). Temporal profiling of plasma cytokines, chemokines and growth factors from mild, severe and fatal COVID-19 patients. Sig Transduct Target Ther. 5, 100 10.1038/s41392-020-0211-1 PMC7303571 32561706

[B161] Yale Impact research team Takahashi T. Ellingson M. K. Wong P. Israelow B. Lucas C. (2020). Sex differences in immune responses that underlie COVID-19 disease outcomes. Nature. 588, 315–320. 10.1038/s41586-020-2700-3 32846427 PMC7725931

[B162] Yasui F. Kai C. Kitabatake M. Inoue S. Yoneda M. Yokochi S. (2008). Prior immunization with severe acute respiratory syndrome (SARS)-Associated coronavirus (SARS-CoV) nucleocapsid protein causes severe pneumonia in mice infected with SARS-CoV. J. Immunol. 181, 6337–6348. 10.4049/jimmunol.181.9.6337 18941225

[B163] Yoshikawa N. Yoshikawa T. Hill T. Huang C. Watts D. M. Makino S. (2009a). Differential virological and immunological outcome of severe acute respiratory syndrome coronavirus infection in susceptible and resistant transgenic mice expressing human angiotensin-converting enzyme 2. J. Virol. 83, 5451–5465. 10.1128/JVI.02272-08 19297479 PMC2681954

[B164] Yoshikawa T. Hill T. Li K. Peters C. J. Tseng C. T. (2009b). Severe acute respiratory syndrome (SARS) coronavirus-induced lung epithelial cytokines exacerbate SARS pathogenesis by modulating intrinsic functions of monocyte-derived macrophages and dendritic cells. J. Virol. 83, 3039–3048. 10.1128/JVI.01792-08 19004938 PMC2655569

[B165] Yoshikawa T. Hill T. E. Yoshikawa N. Popov V. L. Galindo C. L. Garner H. R. (2010). Dynamic innate immune responses of human bronchial epithelial cells to severe acute respiratory syndrome-associated coronavirus infection. PLoS ONE. 5, e8729 10.1371/journal.pone.0008729 20090954 PMC2806919

[B166] Young B. E. Ong S. W. X. Ng L. F. P. Anderson D. E. Chia W. N. Chia P. Y. (2020). Viral dynamics and immune correlates of coronavirus disease 2019 (COVID-19) severity. Clinical Infectious Diseases. [Epub ahead of print]. 10.1093/cid/ciaa1280 PMC7499509 32856707

[B167] Yen Y. T. Liao F. Hsiao C. H. Kao C. L. Chen Y. C. Wu-Hsieh B. A. (2006). Modeling the early events of severe acute respiratory syndrome coronavirus infection in vitro. J. Virol. 80, 2684–2693. 10.1128/JVI.80.6.2684-2693.2006 16501078 PMC1395447

[B168] Yu P. Qi F. Xu Y. Li F. Liu P. Liu J. (2020). Age‐related rhesus macaque models of COVID‐19. Anim Models Exp Med. 3, 93–97. 10.1002/ame2.12108 PMC7167234 32318665

[B169] Wan Y. Shang J. Graham R. Baric R. S. Li F. (2020). Receptor recognition by the novel coronavirus from wuhan: an analysis based on decade-long structural studies of SARS coronavirus. J. Virol. 94, e00127-20 10.1128/JVI.00127-20 31996437 PMC7081895

[B170] Wang D. Hu B. Hu C. Zhu F. Liu X. Zhang J. (2020). Clinical characteristics of 138 hospitalized patients with 2019 novel coronavirus-infected pneumonia in wuhan, China. J. Am. Med. Assoc. 323, 1061 10.1001/jama.2020.1585 PMC7042881 32031570

[B171] Wang W. Chen S. Liu I.-J. Kao C. Kao H. Chiang B. (2004). Temporal relationship of viral load, ribavirin, interleukin (IL)-6, IL-8, and clinical progression in patients with severe acute respiratory syndrome. Clin. Infect. Dis. 39, 1071–1075. 10.1086/423808 15472864 PMC7107918

[B172] Woolhouse M. Gaunt E. (2007). Ecological origins of novel human pathogens. Crit. Rev. Microbiol. 33, 231–242. 10.1080/10408410701647560 18033594

[B173] Zaki A. M. van Boheemen S. Bestebroer T. M. Osterhaus A. D. Fouchier R. A. (2012). Isolation of a novel coronavirus from a man with pneumonia in Saudi Arabia. N. Engl. J. Med. 367, 1814–1820. 10.1056/NEJMoa1211721 23075143

[B174] Zhang X. Tan Y. Ling Y. Lu G. Liu F. Yi Z. (2020). Viral and host factors related to the clinical outcome of COVID-19. Nature. 583, 437–440. 10.1038/s41586-020-2355-0 32434211

[B175] Zhang Y. Li J. Zhan Y. Wu L. Yu X. Zhang W. (2004). Analysis of serum cytokines in patients with severe acute respiratory syndrome. Infect. Immun. 72, 4410–4415. 10.1128/IAI.72.8.4410-4415.2004 15271897 PMC470699

[B176] Zheng S. Fan J. Yu F. Feng B. Lou B. Zou Q. (2020a). Viral load dynamics and disease severity in patients infected with SARS-CoV-2 in Zhejiang province, China, January-March 2020: retrospective cohort study. BMJ. 369, m1443 10.1136/bmj.m1443 32317267 PMC7190077

[B177] Zheng Z. Peng F. Xu B. Zhao J. Liu H. Peng J. (2020b). Risk factors of critical & mortal COVID-19 cases: a systematic literature review and meta-analysis. J. Infect. 81, e16–e25. 10.1016/j.jinf.2020.04.021 PMC7177098 32335169

[B178] Zhou F. Yu T. Du R. Fan G. Liu Y. Liu Z. (2020a). Clinical course and risk factors for mortality of adult inpatients with COVID-19 in Wuhan, China: a retrospective cohort study. Lancet. 395, 1054–1062. 10.1016/S0140-6736(20)30566-3 32171076 PMC7270627

[B179] Zhou P. Yang X. L. Wang X. G. Hu B. Zhang L. Zhang W. (2020b). A pneumonia outbreak associated with a new coronavirus of probable bat origin. Nature. 579, 270–273. 10.1038/s41586-020-2012-7 32015507 PMC7095418

[B180] Zhou Z. Ren L. Zhang L. Zhong J. Xiao Y. Jia Z. (2020c). Heightened innate immune responses in the respiratory tract of COVID-19 patients. Cell Host Microbe. 27, 883–890.e2. 10.1016/j.chom.2020.04.017 32407669 PMC7196896

[B181] Zhu N. Zhang D. Wang W. Li X. Yang B. Song J. (2020). A novel coronavirus from patients with pneumonia in China, 2019. N. Engl. J. Med. 382, 727–733. 10.1056/NEJMoa2001017 31978945 PMC7092803

